# Aged Breast Extracellular Matrix Drives Mammary Epithelial Cells to an Invasive and Cancer‐Like Phenotype

**DOI:** 10.1002/advs.202100128

**Published:** 2021-10-07

**Authors:** Gokhan Bahcecioglu, Xiaoshan Yue, Erin Howe, Ian Guldner, M. Sharon Stack, Harikrishna Nakshatri, Siyuan Zhang, Pinar Zorlutuna

**Affiliations:** ^1^ Department of Aerospace and Mechanical Engineering University of Notre Dame Notre Dame IN 46556 USA; ^2^ Harper Cancer Research Institute University of Notre Dame Notre Dame IN 46556 USA; ^3^ Department of Biological Sciences University of Notre Dame Notre Dame IN 46556 USA; ^4^ Department of Chemistry and Biochemistry University of Notre Dame Notre Dame IN 46556 USA; ^5^ Department of Surgery School of Medicine Indiana University Indianapolis IN 46202 USA; ^6^ Department of Biochemistry and Molecular Biology School of Medicine Indiana University Indianapolis IN 46202 USA; ^7^ Bioengineering Graduate Program University of Notre Dame Notre Dame IN 46556 USA

**Keywords:** aging, breast cancer, epithelial‐mesenchymal transition, extracellular matrix, lysyl oxidase

## Abstract

Age is a major risk factor for cancer. While the importance of age related genetic alterations in cells on cancer progression is well documented, the effect of aging extracellular matrix (ECM) has been overlooked. This study shows that the aging breast ECM alone is sufficient to drive normal human mammary epithelial cells (KTB21) to a more invasive and cancer‐like phenotype, while promoting motility and invasiveness in MDA‐MB‐231 cells. Decellularized breast matrix from aged mice leads to loss of E‐cadherin membrane localization in KTB21 cells, increased cell motility and invasion, and increased production of inflammatory cytokines and cancer‐related proteins. The aged matrix upregulates cancer‐related genes in KTB21 cells and enriches a cell subpopulation highly expressing epithelial‐mesenchymal transition‐related genes. Lysyl oxidase knockdown reverts the aged matrix‐induced changes to the young levels; it relocalizes E‐cadherin to cell membrane, and reduces cell motility, invasion, and cytokine production. These results show for the first time that the aging ECM harbors key biochemical, physical, and mechanical cues contributing to invasive and cancer‐like behavior in healthy and cancer mammary cells. Differential response of cells to young and aged ECMs can lead to identification of new targets for cancer treatment and prevention.

## Graphical abstract

Aging leads to reduced collagen XV and V, and increased stiffness, cytokine content, collagen and fiber thickness, and curvature in the breast tissue. The aged extracellular matrix, without the cellular components, predisposes the normal and cancer cells to a more invasive phenotype, by changing their gene expression profile. Lysyl oxidase knockdown reverses all the effects.

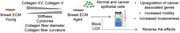

## 1 Introduction

Cancer incidence increases dramatically with age,^[^ ^1^ ^]^ suggesting that aging may promote tumorigenesis. This clinical observation has so far been attributed to effects of aging on the genetic makeup of cells,^[^ ^2^, ^3^ ^]^ and therefore aging‐associated changes in tissues have conventionally been investigated at the cell level.^[^ ^4^, ^5^, ^6^ ^]^ While the effect of aging‐associated dysregulation of the cellular machinery on cancer initiation is well documented, the effect of aging‐associated changes in the microenvironment, specifically the extracellular matrix (ECM), is overlooked. It is known that neoplastic transformation of rat liver epithelial cells leads to higher rates of tumor formation when cells are transplanted into livers of aged rats compared to young,^[^ ^7^ ^]^ suggesting that the aged microenvironment plays important roles in tumor initiation and progression. However, whether and how the aged ECM contributes to cancer initiation and progression is not known.

Even slight differences in the biochemical composition, stiffness, and structure of the ECM may lead to a significant difference in cellular response.^[^ ^8^, ^9^, ^10^ ^]^ For instance, collagen I promotes epithelial‐mesenchymal transition (EMT),^[^ ^11^, ^12^ ^]^ while collagen XV prevents it.^[^ ^12^ ^]^ In the aged microenvironment, collagen production decreases and the ECM integrity is lost, leading to a greater invasive ability of tumor cells.^[^ ^1^, ^13^ ^]^ Decrease in fiber thickness is another age‐related alteration in the ECM that could be contributing to metastasis.^[^ ^14^ ^]^ On the other hand, substrate stiffness is required for the transformation of normal breast epithelial cells into tumor precursors^[^ ^15^ ^]^ and cancer cells on stiff matrices block adipocyte differentiation and maturation.^[^ ^16^ ^]^ Conversely, culturing malignant progenitors on soft substrates reverts them to normal epithelial cells.^[^ ^17^ ^]^ The effect of aged ECM on EMT and invasiveness of normal epithelial cells, however, has not been investigated.

Despite the fact that breast cancer is one of the most common and widely studied cancer types, there is a dearth of research on age‐related alterations in the ECM of the mammary gland. Here, we provide a full characterization of the structural, mechanical, and biochemical changes that occur in the mouse breast ECM upon aging, and investigate the response of normal and cancerous human mammary epithelial cells to aged decellularized breast matrices. This study is the first to show that the aged ECM drives EMT‐like and invasive behavior in normal epithelial cells as well as cancer cells, which indicates that the aged microenvironment contains components that lead to tumor initiation and progression. This study is also the first to report the aging‐associated changes in the breast ECM, which could pave the way for new therapeutic options as well as engineering better tumor models.

## 2 Results

### 2.1 Aging Leads to Thicker Collagen Fibers, Greater Modulus, and Altered Biochemical Composition in the Breast

**Figure 1:**
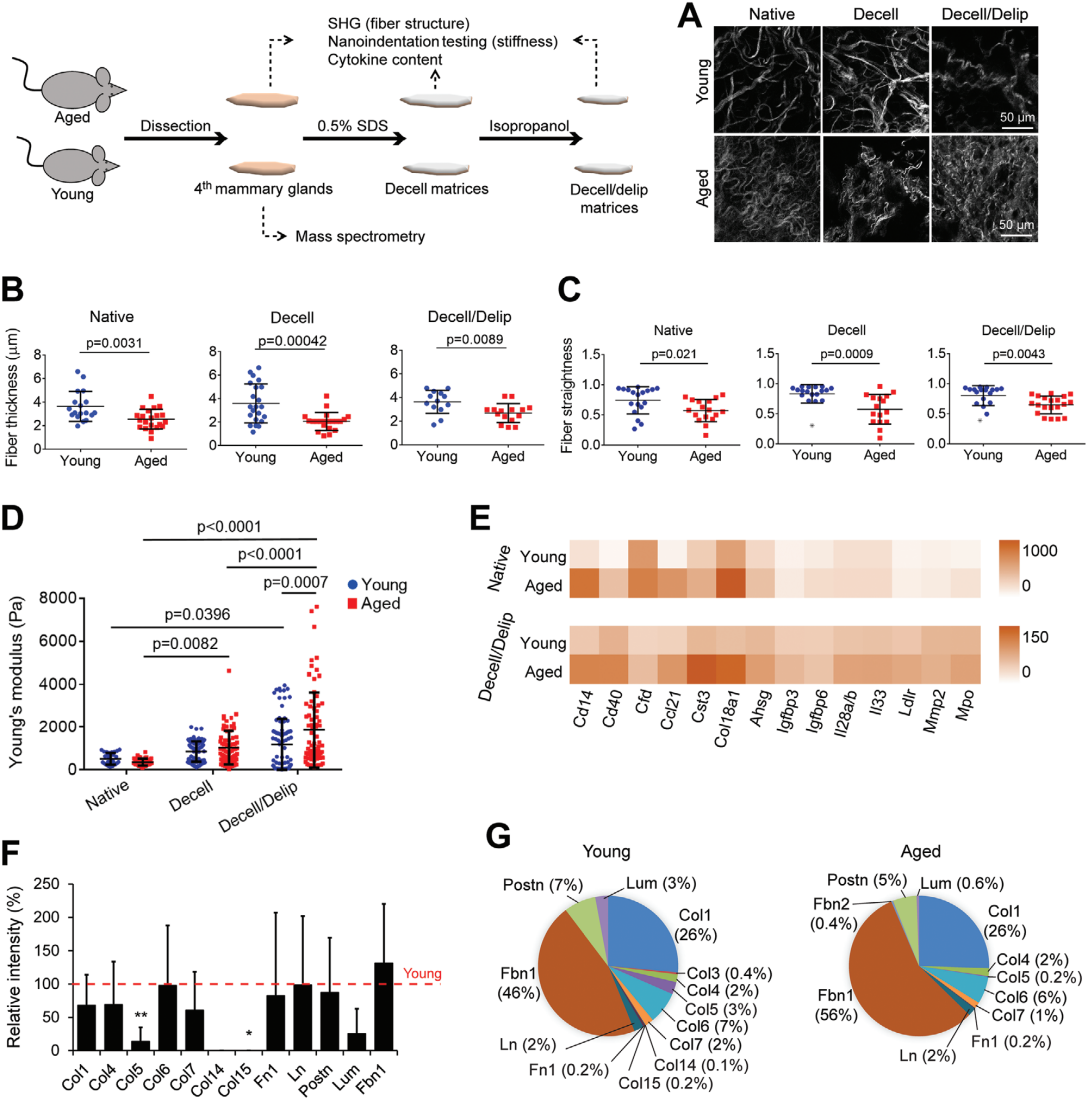
Aging leads to thinner and curvier collagen fibers, denser collagen mesh, increased stiffness and cytokine levels, and decreased structural protein levels in the breast. A) Representative second harmonic generation (SHG) images showing the collagen fiber structure. B) Fiber diameter and C) fiber straightness as quantified from the SHG images in (A) with Fiji software. *n* = 3 samples (each from a different mouse) and *n* = 4–8 fibers/sample were analyzed. D) Elastic modulus as determined with nanoindentation testing. *n* = 5 tissues/group (each tissue from a different mouse). Each sample was tested on 5–20 different locations. E) Cytokine profiling of the native tissue and decell/delip matrix as quantified by dot blot‐based immunoassay. *n* = 3 pooled samples (each from a different mouse). Results representative of two independent experiments. See also Figure S1C and Table S1, Supporting Information. F,G) Mass spectrometric analysis showing the most abundant proteins in the native tissues. F) Signal intensity of the proteins in the aged matrix relative to that in the young (red line). *p < 0.05, and **p < 0.01. G) Percentages of proteins present in the matrices. *n* = 3 young and 5 aged samples (each from a different mouse). Data are presented as the mean ± SD. Student's *t*‐test was applied for (B), (C), and (F); and one‐way ANOVA followed by Tukey's post hoc for (D).

First, we verified that our decellularization (cell removal) and delipidation (fat removal) protocol removed most of the DNA in the tissues, yielding <50 ng mg^−1^ dry tissue weight (Figure S1A, Supporting Information). Then, to understand the age‐related changes in the breast ECM, we characterized tissues from young (3–6 months old) and aged (22–25 months old) mice before (native tissue) and after decellularization (decell matrix) and delipidation (decell/delip matrix). It is known that breast becomes less dense with aging due to increased fat content.^[^ ^18^, ^19^ ^]^ Here, we observed a lumpy, patchy, and more compact collagen network, and thinner (≈30% decrease in collagen fiber diameter, *p* < 0.01) and curvier (≈24% decrease in fiber straightness, *p* < 0.05) collagen fibers in the aged tissue, regardless of the decellularization/delipidation status (**Figure** **1A**–C, and Figure S1B, Supporting Information).

Stiffness changes with age in various tissues;^[^ ^20^ ^]^ however, the effect of aging on mechanical properties of the breast has not been studied, although ultrasound elastography based measurements on patients have indicated a slight increase in stiffness of the echogenic homogeneous fibroglandular tissues in breast upon aging.^[^ ^19^ ^]^ Here, using nanoindentation testing we found that the elastic modulus of the native breast decreased with age (young: 509 ± 275 Pa, and aged: 356 ± 162 Pa) (Figure 1D). However, the modulus of aged matrix (1867 ± 1765 Pa) was greater than the young (1180 ± 1226 Pa) after removal of cells and fat (*p* < 0.0089). Decellularization and delipidation increased the modulus, and this increase was more pronounced with the aged matrix (4.3 fold increase, *p* < 0.0001) compared to the young (1.3 fold increase, *p* = 0.04). Considering that decellularization using 0.5% sodium dodecyl sulfate (SDS) does not significantly change the mechanical properties of non‐fatty tissues,^[^ ^21^, ^22^ ^]^ the significant increase in the modulus of the breast tissues after decellularization/delipidation would mainly be due to removal of fatty components. Our results suggest that aged tissues contained more fatty components, yet the fibrous component of their ECM is stiffer.

The effect of aging on the matrisome of mammary gland has not been well‐studied, but cytokine levels are known to increase with age in other tissues,^[^ ^1^, ^5^, ^23^ ^]^ and collagen I and IV, laminin 1 (Ln), and periostin (Postn) to decrease.^[^ ^24^, ^25^, ^26^ ^]^ Our data show that the levels of Cd14, Cd40, Ccl21, complement factor D (Cfd), endostatin (Col18a1), cystatin C (Cst3), myeloperoxidase (Mpo), and fetuin A (Ahsg) were elevated with age, and this trend was preserved after decellularization/delipidation, although total cytokine levels decreased compared to native tissue (Figure 1E, and Figure S1C and Table S1, Supporting Information). We also tested the release of these cytokines from the decellularized matrices. Expectedly, after 7 days of incubation in cell‐ and serum‐free media, the aged decellularized matrix released higher levels of cytokines than the young matrix, including Cd40, Cst3, Col18a1, and Mpo, as well as Gas6, Mmp3, Reg3G, and Vegf (Figure S4D, Supporting Information).

Conversely, the amounts of structural ECM proteins was reduced with age except for fibrillin 1 (Fbn1), which increased, and particularly remarkable were the decreases in collagens V (*p* = 0.007) and XV (*p* = 0.044) (Figure 1F). On the other hand, Fbn1 (46–56% of the total structural protein counts) and collagen I (Col1, 26% of the total protein counts) constituted the majority of the detectable proteins; and the proportion of Fbn1 within the structural ECM proteins increased with age (Figure 1G). The decrease in collagen I was also verified with immunostaining (Figure S1D, Supporting Information) and western blotting (Figure S1E, Supporting Information). The proportion of lumican (Lum) decreased with age, while those of other proteins stayed relatively stable. Additionally, hematoxylin and eosin, as well as Masson's trichrome staining of the native tissues showed a reduction in the size of epithelium portion with aging (Figure S1F, Supporting Information), as expected,^[^ ^27^ ^]^ which could be one reason for the reduced protein levels in the aged tissues. Collectively, these data show that the majority of the structural proteins, except for Fbn1, decrease with age, while the levels of most cytokines were elevated.

### 2.2 Aged Microenvironment Leads to EMT‐Like and Invasive Behavior in Normal Mammary Epithelial Cells

**Figure 2:**
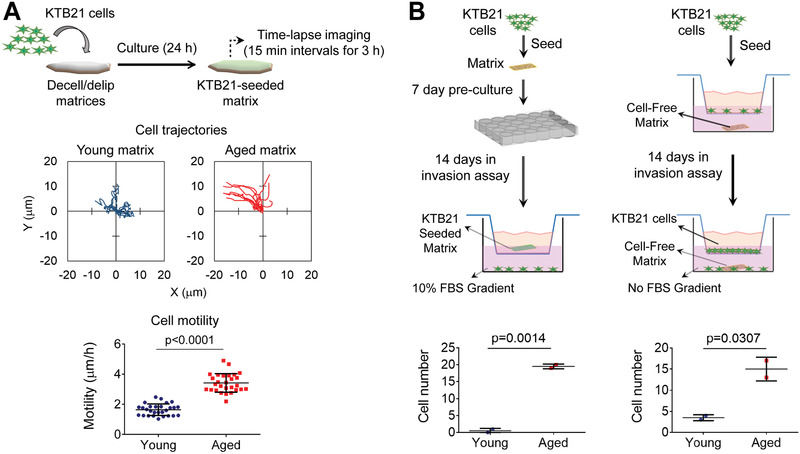
Aged breast ECM induces KTB21 cell migration and invasion. A) Cell migration on matrices calculated from time‐lapse images taken at 15 min intervals for 3 h. Top: cell trajectories, bottom: motility. Also see Movies S1 and S2. B) Cell invasion through transwell inserts. Left: invaded cell number against FBS gradient. *n* = 2 matrices. Right: invaded cell number towards cell‐free matrices in the bottom chamber. *n* = 2 matrices. Data are presented as the mean ± SD. Statistical tests: two‐tailed student's *t*‐test.

We investigated the effect of aged ECM on the migration and invasion behavior of KTB21 normal human mammary basal epithelial cells, which we established previously.^[^ ^28^ ^]^ Remarkably, cell motility was higher on the aged matrix than the young (*p* < 0.0001) (**Figure** **2A**, and Movies S1 and S2, Supporting Information). For invasion, we used two approaches. In the first, we seeded the cells on the matrices and pre‐incubated for 7 days, followed by a 14‐day incubation in transwell inserts (upper chamber) against a 10% FBS gradient (bottom well) (Figure 2B, left). The number of cells that invaded through the inserts was higher in the presence of aged matrix than young (*p* = 0.0014). The reason for the increased number of invaded cells on the aged matrix could be the higher invasiveness of these cells or the higher motility, which resulted in higher number of cells migrating from the matrix surface to the insert surface. Therefore, in the second approach, we seeded KTB21 cells in transwell inserts and placed the cell‐free matrices in the bottom wells (expecting that the matrices would release cytokines to the media and act as chemoattractants) (Figure 2B, right). After a 14‐day incubation, the number of cells that invaded towards the aged matrix was significantly higher than that of cells invaded towards the young (*p* = 0.0307), indicating that cytokines released from the aged matrices (Figure S1D, Supporting Information) promoted the invasion of cells.

Similar migratory and invasive behavior was observed with the MDA‐MB‐231 breast cancer cells. Cell motility on the aged matrix was greater than that on the young (*p* < 0.0001) (Figure S2A,B, and Movies S3 and S4, Supporting Information). Invading cell count was also higher in the presence of aged matrix (Figure S2C, Supporting Information).

**Figure 3:**
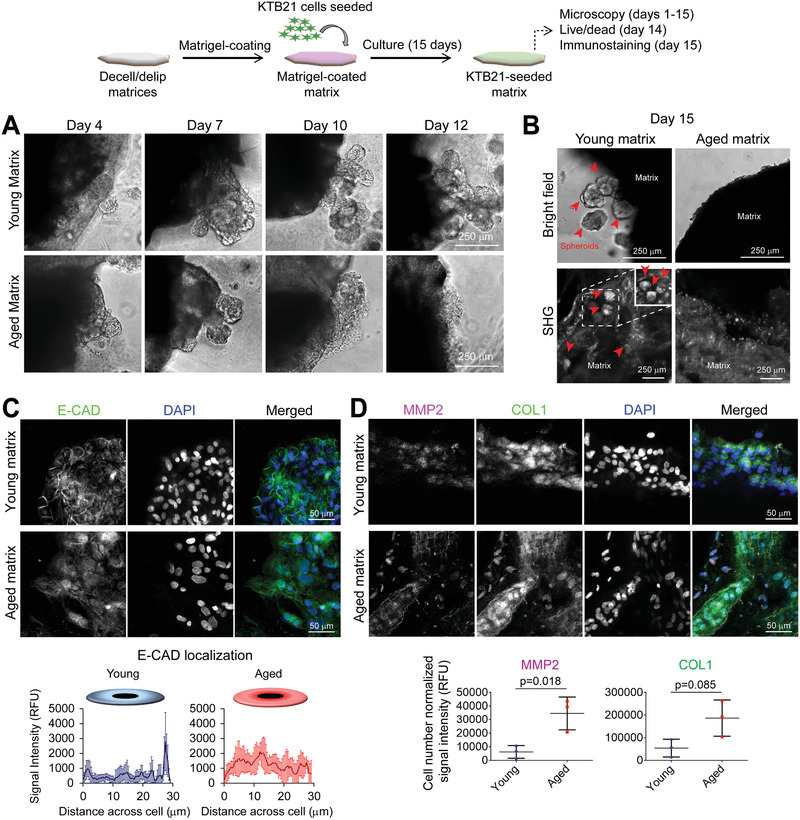
Aged breast ECM leads to deformation of KTB21 cell spheroids and delocalization of E‐CAD from cell membrane to cytosol. A) KTB21 spheroids on the matrices monitored for 12 days. B) Microscopy images showing the spheroid presence on matrices at day 15. Top: bright field and bottom: second harmonic generation (SHG) images. Inset: image at a deeper focal plane of the dashed square. Arrowheads point to spheroids. C,D) E‐CAD, MMP2, and COL1 staining (day 15). C) E‐CAD localization in cells. Top: representative confocal microscopy images of matrices. *n* = 3. Bottom: plot profile of E‐CAD signal intensity across a representative cell. *n* = 6 cells/matrix for young and 9 for aged. D) MMP2 and COL1 staining. Top: representative confocal microscopy images of matrices. Bottom: quantification of the signal intensities. *n* = 3. E‐CAD and COL1: Alexa fluor 488 (green), MMP2: Alexa fluor 647 (magenta). Nucleic acid: DAPI (blue). Quantifications are performed using the Fiji software. Data are presented as the mean ± SD. Statistical test: two‐tailed student's *t*‐test.

Next, we investigated how aging would influence acini formation on the matrices. For this, we coated the matrices with Matrigel, which mimics the basement membrane in the mammary gland, seeded the KTB21 cells on the Matrigel‐coated matrices, and monitored the spheroid formation and stability for 15 days. KTB21 cells formed spheroids both on the young and aged matrices (**Figure** **3A**). Interestingly, while spheroids remained intact on the young matrices for more than 15 days, they started to deform on the aged matrices at day 10 of incubation and disintegrated (dispersed or flattened) by day 15 (Figure 3A,B). Furthermore, the spheroids on the aged matrices were less spherical (lower circularity [*p* < 0.0001], and greater aspect ratio [*p* < 0.005]) (Figure S3A, Supporting Information). Cell viability was high (≈85%) on both matrices, showing that deformation of the spheroids was not due to cell death (Figure S3A, Supporting Information).

The disruption of spheroids on the aged matrices could be due to reduced cell–cell interactions and/or higher rate of matrix degradation on these matrices. Therefore, at day 15 of culture we stained the KTB21 cells seeded on matrices for E‐cadherin (E‐CAD) (to assess cell–cell interactions), matrix metalloproteinase 2 (MMP2) (to assess matrix degradation), and COL1 (to assess matrix remodeling). Strikingly, while E‐CAD was localized to cell membrane on the young matrix, little membrane localization was observed on the aged matrix (Figure 3C and Figure S3B, Supporting Information). Additionally, higher amounts of MMP2 (*p* = 0.018) and COL1 (*p* = 0.085) were deposited on the aged matrices (Figure 3D, and Figure S3C,D, Supporting Information). The collagen in the matrices could be distinguished from the collagen deposited by cells (Figure S3D,E, Supporting Information). These results showed that cell–cell adhesion might be reduced, and matrix degradation might be increased on the aged matrix, which both might contribute to the disintegration of the spheroids. Additionally, cells, especially those on the aged matrix, produced their own ECM, showing that the ECM was remodeled.

### 2.3 Aging Microenvironment Leads to Upregulation of Cancer‐Associated Genes and Enrichment of a Cell Cluster Defined by EMT Transcriptome

**Figure 4:**
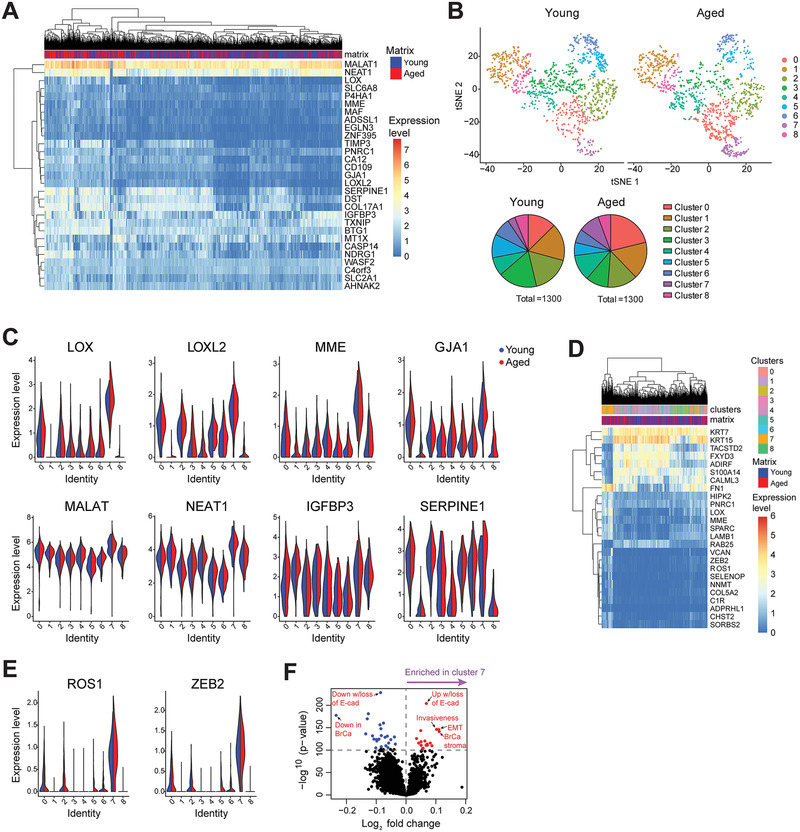
scRNA‐seq of KTB21 cells incubated for 20 days on young and aged matrices reveals upregulation of invasion and migration related genes and enrichment of an invasive sub‐population on the aged matrices. A) Genes significantly upregulated in cells on the aged matrix. B) Clustering of cells on matrices after down‐sampling. Top: t‐SNE showing the transcriptional differences between cells and clusters within each matrix type (closer cells have similar transcriptomes). Bottom: pie charts comparing the cell cluster sizes on a specific matrix type based on the cell numbers belonging to a specific cluster. C) Expression of the invasion and migration related genes that have upregulated on the aged matrices in cell clusters. D) Genes significantly differentially expressed in cluster 7 cells compared to cells in other clusters. E) Genes significantly upregulated in cluster 7 cells. F) ssGSEA showing enrichment of invasion and EMT‐associated gene sets in cluster 7 cells. *n* = 1300 cells/matrix type, pooled from 2 independent biological replicates. Adjusted *p* < 0.05.

To further examine the phenotypic changes we observed in normal epithelial cells, we performed single‐cell RNA‐sequencing (scRNA‐seq) to investigate how 20‐day culture on aged matrix impacts the transcriptome. Two independent biological replicates were prepared from each of the KTB21 cell‐seeded young and aged matrices, with 1470 and 571 high‐quality single cell transcriptomes identified in the young samples, and 1098 and 414 in the aged (Figure S4A, Supporting Information). For analysis, the replicates were combined, and randomly made subset to include 1300 in each condition. The distribution of cells was largely similar between conditions (Figure S4B, Supporting Information). We first examined expression of known markers of the KTB21 basal mammary epithelial cells to confirm maintenance of the basal phenotype. A large proportion (70%) of the cells expressed the basal epithelial cell markers, *KRT5* and *KRT14*, and a smaller subset expressed the luminal markers, *KRT8* and *KRT18* (Figure S4C, Supporting Information), in line with previous characterization.^[^ ^28^ ^]^ Analysis of all 2600 cells revealed that 18 genes were downregulated in cells on aged matrix, including *HSPA8* and *CCND1*, which are related with cell proliferation,^[^ ^29^, ^30^ ^]^ and *NME1* and *PHLDA1*, which inhibit metastasis,^[^ ^31^, ^32^ ^]^ while 28 genes were upregulated including *NDRG1*, *EGLN3*, *P4HA1*, *LOX, LOXL2, MME, GJA1, MALAT1, NEAT1, TIMP3, IGFBP3*, and *SERPINE1*, which are involved in EMT, and cell migration and invasion (**Figure** **4A** and Table S2, Supporting Information, adjusted *p*‐value < 0.05).^[^ ^33^, ^34^, ^35^, ^36^ ^]^ Gene ontology (GO) analysis also showed negative regulation of cell division, growth, and adhesion in cells on the aged matrix, and positive regulation of epithelial cell migration, angiogenesis/vascularization, and matrix remodeling (Figure S4D, Supporting Information).

Consistent with analysis of all cells (Figure S4B, Supporting Information), cluster analysis identified 9 transcriptional clusters. The top 10 genes defining each cluster of cells are shown in Figure S5D, Supporting Information. Cells cultured on both young and aged matrices were represented in each cluster, suggesting that neither matrix induced the formation of a new sub‐population of cells (Figure 4B, top). However, culture on aged matrices led to an enrichment of cells in cluster 0 (275 cells out of 1300 on aged matrix were identified as cluster 0 cells, compared to 164 cells out of 1300 on young matrix) and cluster 7 (123 cells on aged matrix, and 44 cells on young) (Figure 4B, bottom). Interestingly, the markers of cluster 7 largely overlapped with genes that were upregulated in the aged microenvironment; 21 out of the 28 genes that were upregulated in the aged microenvironment, were also expressed in cluster 7 cells at significantly higher levels than any other cluster (Figure 4C, and Tables S2 and S3, Supporting Information).

Closer examination of the transcriptional differences separating cluster 7 from the remaining cells revealed loss of expression of the epithelial *TACSTD2* and *KRT15*, and the adipogenic *ADIRF* (Figure 4D and Table S2, Supporting Information). More importantly, cluster 7 cells were defined by increased expression of a number of genes involved in EMT‐like processes, including *ZEB2*, *LOX*, *ROS1*, *FN1*, *VCAN*, and *SPARC*. Specifically expression of *ZEB2* and *ROS1* was significantly higher in cluster 7 cells than others (Figure 4E). Further examination of the genes defining cluster 7 using single sample gene set enrichment analysis (ssGSEA) revealed enrichment of invasive and EMT‐associated gene sets in cluster 7 cells (Figure 4F). Taken together, scRNA‐seq suggests the existence of a subpopulation of cells that is pre‐invasive, or predisposed to undergo EMT in normal mammary epithelial cells, which is enriched when they engage with aged matrix compared to young.

### 2.4 LOX Knockdown Reverses the Aged Matrix‐Induced Changes in Cell Phenotype, E‐CAD Localization, Protein Expression, and Cell Migration and Invasion

Increased mechanical stress on epithelial cells leads to internalization of E‐CAD to the cytosol,^[^ ^37^ ^]^ and lysyl oxidase (LOX), an enzyme that crosslinks collagen and elastin in the ECM and stiffens it, may prevent the localization of E‐CAD to the cell membrane.^[^ ^38^ ^]^ As we showed that E‐CAD was delocalized in cells on aged matrix, and as *LOX* was highly expressed in cells on the aged matrices both in bulk and in cluster 7 cells, we investigated the effect of *LOX* siRNA treatment on E‐CAD expression and localization, spheroid formation, cytokine production, and cell migration and invasion. First, we verified higher LOX (31% ± 17%, *p* = 0.034) and E‐CAD (8%) protein expression on the aged matrices (Figure S5A, Supporting Information). Then, we knocked down LOX by treatment with *LOX* siRNA. Successful knockdown was shown with reduced mRNA (Figures S5B,C, Supporting Information) and protein (Figure S5D, Supporting Information) levels after *LOX* siRNA treatment of cells on culture plate. Interestingly, E‐CAD protein production was also reduced with *LOX* siRNA treatment. LOX active protein levels were shown to decrease after treatment of cells on the young (by 22% ± 31%) and aged (by 30% ± 13%, *p* = 0.076) matrices with *LOX* siRNA (Figure S5E, Supporting Information). The results also confirmed that LOX is produced at higher levels on the aged matrices (30%).

**Figure 5:**
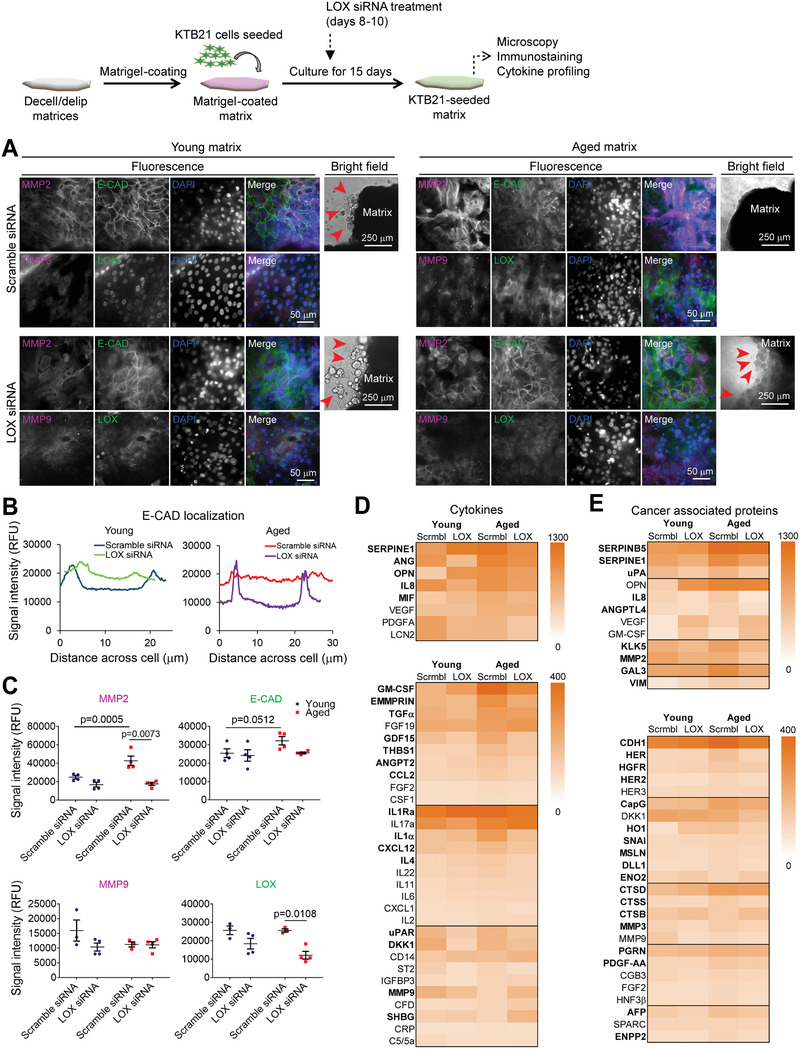
LOX knockdown reverses the phenotypic and biochemical changes in KTB21 cells induced by the aged microenvironment. A) Microscopy images showing MMP2, MMP9, E‐CAD, and LOX expression, and spheroid stability on matrices (day 15). siRNAs applied between days 8–10. Fluorescence microscopy images showing MMP2 and MMP9 (magenta, Column 1), E‐CAD and LOX (green, Column 2), and nucleic acid (DAPI, blue, Column 3) staining, and merged images (Column 4). Bright field images (Column 5) showing the spheroids on matrices. *n* = 4. Left: young matrices, and right: aged matrices. Upper panel: scramble siRNA, and lower panel: *LOX* siRNA treated matrices. Arrowheads show spheroids. Results representative of two independent experiments. B) Plot profile of E‐CAD signal intensity along the diameter of representative cells in (A) showing E‐CAD localization in the cells. *n* = 4 images/matrix, and 8–10 cells/image. C) Cell number‐normalized signal intensities of MMP2, MMP9, E‐CAD, and LOX as quantified from (A). *n* = 4 matrices. Representative of three independent experiments. D,E) Heat map showing the dot blot‐based cytokine profiling of the cells on matrices (day 15). D) Cytokines and E) cancer‐associated proteins expressed by the cells. *n* = 3 pooled samples. Also see Tables S4 and S5, Supporting Information. Quantifications were performed using the Fiji software. Data are presented as the mean ± SD. Statistical tests: one‐way ANOVA followed by Tukey's post hoc.

*LOX* siRNA treatment prevented the disintegration of KTB21 spheroids (red arrowheads) on the aged matrix until day 15 of culture, which otherwise disperse completely before day 15 (usually around day 13), and increased the number of spheroids on the young matrix (**Figure** **5A**). Scramble siRNA treatment did not show any difference from no treatment group (Figure 5A versus Figure 3B); spheroids on the aged matrix had disintegrated completely by day 15, while those on the young matrix stayed intact. Additionally, *LOX* siRNA treatment reversed the delocalization of E‐CAD in cells on the aged matrix, leading to membrane‐localized E‐CAD expression, while slightly delocalizing E‐CAD on the young matrix (Figure 5A,B). Immunostaining also verified that LOX protein expression was reduced on the aged matrix after *LOX* siRNA treatment (*p* = 0.0108) (Figure 5A,C). Interestingly, MMP2 (*p* = 0.0073), but not MMP9, was significantly reduced on the aged matrix after *LOX* siRNA treatment. These results are in line with the Genotype‐Tissue Expression (GTEx) breast tissue and The Cancer Genome Atlas (TCGA) breast invasive carcinoma datasets, which show a correlation between *LOX* and *MMP2* (*p* < 10^–6^) expression (Figure S6A, Supporting Information), but not between *LOX* and *CDH1* (E‐CAD) (Figure S6B, Supporting Information) or *LOX* and *MMP9* (Figure S6C, Supporting Information).

We next performed cytokine profiling for the KTB21 cells seeded on decell/delip matrices before and after siRNA treatment. Most of the cytokines, especially the uPA/uPAR mediators SERPINE1, ANG, OPN, and uPAR, the pro‐inflammatory IL8, MIF, GM‐CSF, TNF*α*, IL1, and IL4, and the MMP enhancer EMMPRIN were produced at higher levels on the aged matrix, but were reduced to the young matrix levels after *LOX* siRNA treatment (Figure 5D, and Figure S7A and Table S4, Supporting Information). Interestingly, some factors like FGF‐19, IL17a, CD14, MMP9, and SHBG increased in the aged microenvironment after *LOX* siRNA treatment.

We also performed cancer‐associated protein profiling to show how aging environment influenced the expression of some cancer‐associated proteins. Similar to the cytokine profiling results, most of the cancer‐associated proteins, such as ANGPTL4, KLK5, GAL3, VIM, HERs, CAPG, HO, CTSD, and AFP were produced at higher levels on the aged matrix, but were reduced to young matrix levels after *LOX* siRNA treatment (Figure 5E, and Figure S7B and Table S5, Supporting Information). SERPINB5 (Maspin) and VEGF were produced at high levels on the aged matrix, and while VEGF increased upon *LOX* siRNA treatment, SERPINB5 did not change.

Cytokine profiles were in consistence with the scRNA‐seq data, as GO analysis showed that biological processes like plasminogen activation and angiogenesis or vascularization were activated in cells on the aged matrix (Figure S4D, Supporting Information).

**Figure 6:**
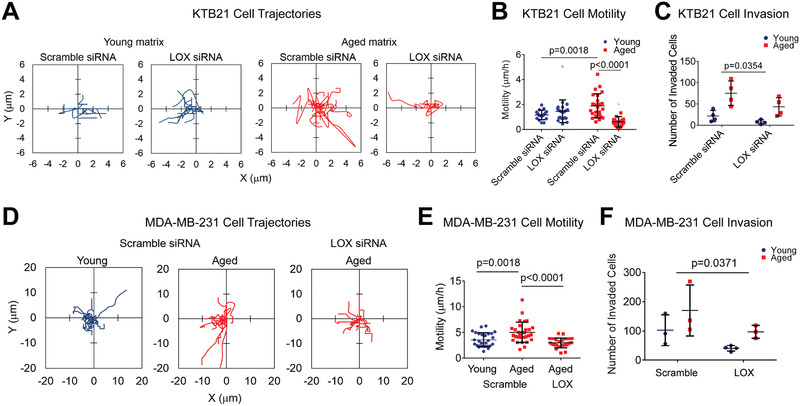
Migration and invasion of KTB21 and MDA‐MB‐231 cells on matrices are reduced after *LOX* siRNA treatment. A–C) KTB21 cells, and D,E) MDA‐MB‐231 cells. A,B) Migration of KTB21 cells. A) Cell trajectories and B) the calculated motility results. siRNAs were applied on cells for 48 h before seeding on matrices. Time‐lapse images were taken at 15 min intervals for 2 h. Outliers are shown as light color dots. Also see Movies S5–S8, Supporting Information. C) Invaded cell number after siRNA treatment. Cell‐seeded matrices were placed in transwell inserts at day 7 and incubated against a 10% FBS gradient until day 21. siRNAs were applied between days 8–10, and also between days 15–17. D,E) Migration of MDA‐MB‐231 cells. D) Cell trajectories and E) motility. siRNAs were applied on cells for 48 h before seeding on matrices. Time‐lapse images taken at 15 min intervals for 3 h. Also see Movies S9–S11, Supporting Information. F) Cell invasion after siRNA treatment. Cells were seeded on the matrices, pre‐incubated for 5 days, treated with siRNAs between days 5–7. Invasion assay was started at day 7 of culture and applied for 4 days (until day 11). *n* = 3–4 matrices. Quantifications were performed using the Fiji software. Data are presented as the mean ± SD. Statistical tests: one‐way ANOVA followed by Tukey's post hoc for (B) and (E), and two‐way ANOVA for (C) and (F).

Additionally, we examined the effect of *LOX* knockdown on cell migration and invasion. While *LOX* knockdown showed no effect on the migration of KTB21 cells on the young matrix, it led to a significant reduction in cell migration on the aged matrix (**Figures** **6A** and 6B, and Movies S5–S8, Supporting Information). Cell motility was even below the level of that on the young matrix. A similar finding was observed with the cell invasion results. The number of invading cells after a 14‐day incubation in the transwell inserts decreased significantly upon *LOX* knockdown on both the young and the aged matrices (*p* < 0.0354, two‐way analysis of variance [ANOVA]) (Figure 6C and Figure S8, Supporting Information).

Similar migratory and invasive behavior was observed with the MDA‐MB‐231 breast cancer cells. Motility of the scramble siRNA‐treated cells was significantly greater on the aged matrix than on the young (*p* = 0.0018), and *LOX* siRNA treatment reduced the motility to below the young matrix level (*p* < 0.0001) (Figures 6D and 6E, and Movies S9–S11, Supporting Information). Cell invasion was also reduced on the young and aged matrices after *LOX* knockdown (*p* < 0.0371, two‐way ANOVA) (Figure 6F).

Finally, we searched the TCGA invasive breast carcinoma data set for the survival of breast cancer patients relative to LOX expression level. We found that high LOX expression significantly reduces survival for luminal cancer patients (Figure S9A, Supporting Information). Luminal cancers are more common in old people than other cancer types, and luminal cancer incidence increases with age.^[^ ^39^ ^]^ In fact, high LOX expression was also associated with poor patient survival in post‐menopausal women, but not in pre‐menopausal women who are expected to be younger (Figure S9B, Supporting Information).

## 3 Discussion

Here we report that aged breast ECM promotes EMT‐like and invasive behavior in normal (KTB21) and cancerous (MDA‐MB‐231) human mammary epithelial cells. Aged microenvironment induces E‐CAD delocalization from cell membrane to cytosol, increases the expression of MMP2, pro‐inflammatory cytokines (IL8, MIF, GM‐CSF, TNF*α*, IL1, and IL4), uPA system components (SERPINE1, ANG, OPN, and uPAR), and several cancer‐related proteins (ANGPTL4, KLK5, MMP3, GAL3, VIM, HERs, CapG, HO, CTSD, and AFP), all involved in cancer progression. Aging ECM also promotes cell motility and invasion by upregulating *LOX*, *LOXL2*, *SERPINE1*, *MME*, *GJA1*, *MALAT*, *NEAT1*, and *IGFBP3* expression, and enriches a subpopulation of cells expressing EMT‐related genes, including *ZEB2*, *ROS1*, *FN1*, *VCAN*, and *SPARC*. Remarkably, *LOX* knockdown leads to re‐localization of E‐CAD to cell membrane, represses the cytokines and oncogenic proteins, and reduces cell motility and invasion on the aged matrix, matching the levels in the aged microenvironment to those in the young.

Dissecting the reasons for the induced invasive and EMT‐like phenotype of the normal and cancerous mammary epithelial cells in the aged microenvironment is difficult, since the ECM is a complex network of proteins and signaling molecules. We show that aging leads to thinner and more wavy collagen fibers, increased Young's modulus, elevated levels of the pro‐inflammatory cytokines (Ccl21, Cd14, Cd40, Cfd), protease inhibitors (Col18a1 and Cst3), and Mpo, as well as decreased levels of structural proteins (Col5 and Col15), each potentially contributing to EMT‐like and invasive phenotype. Additionally, we show that cytokines present in the aged matrix can increase the invasiveness of cells, and along with other biochemical and physical cues, they may promote cell migration.

Collagen structure (density and fiber thickness), stiffness, cytokines, and composition may all play roles in cancer initiation and progression.^[^ ^1^, ^9^, ^14^, ^20^, ^23^, ^34^ ^]^ Here, we highlight the importance of reduced Col15 and Col5 levels, as well as increased stiffness and cytokine levels in the aged microenvironment. Decreased Col15 in the aged matrix, which is a tumor suppressor localized to basement membrane and involved in stabilization of E‐CAD and prevention of its internalization to cytosol,^[^ ^12^ ^]^ could be the reason for the delocalization of E‐CAD in cells on the aged matrix. The reduced Col5 in the aged matrix, which plays a role in fibril assembly,^[^ ^40^ ^]^ could be the reason for the thinner collagen fibers. A previous study has reported lower production of COL5 and COL15 proteins in xenografts created using the more aggressive MDA‐MB‐231‐NM2 cell line compared to the less aggressive MDA‐MB‐231,^[^ ^41^ ^]^ indicating the role of Col5 and 15 in invasive behavior. On the other hand, the stiffer aged matrix might contribute to the higher KTB21 and MDA‐MB‐231 cell motility and invasion,^[^ ^42^, ^43^ ^]^ E‐CAD endocytic internalization,^[^ ^37^ ^]^ LOX and MMP2 expression,^[^ ^44^, ^45^ ^]^ and cytokine and growth factor production,^[^ ^46^, ^47^ ^]^ which in turn could lead to more migratory and invasive behavior. In fact, we showed that cytokines which are present at higher levels in the aged tissue and released to culture media induced cell invasion. Of particular interest were Cd14, which is involved in EMT, tumor invasion and progression, and establishing proinflammatory tumor microenvironment,^[^ ^48^, ^49^, ^50^ ^]^ Ccl21, which has been shown to induce melanoma cell metastasis in mice,^[^ ^51^ ^]^ and Cd40, which is known to induce cell migration.^[^ ^52^ ^]^ Higher stiffness of the aged matrix in combination with the high cytokine content could be the reason for the high proinflammatory and cancer‐associated cytokine and protein production by the epithelial cells.

On the cellular side, we show that although KTB21 cells can form spheroids on both young and aged matrices, spheroids in the aged microenvironment deform and disintegrate after day 10 (usually around day 13) in culture. The disintegration of spheroids in the aged microenvironment could be due to faster degradation of the matrix and/or weaker cell adhesion. Indeed, we show that MMP2 is expressed at higher levels on the aged matrix and E‐CAD is delocalized from cell membrane to cytosol. It is known that MMP2 is expressed at high levels in breast cancer.^[^ ^53^ ^]^ Moreover, E‐CAD internalization to cytosol plays a role in EMT,^[^ ^54^ ^]^ and its localization to cell membrane is disrupted in breast cancer.^[^ ^55^ ^]^ Here, we show for the first time that the aged ECM leads to E‐CAD delocalization.

We next report increased motility and invasion of both normal and cancer cells, as well as upregulated *NDRG1, EGLN3, P4HA1, LOX*, *LOXL2*, *SERPINE1*, *MME*, *GJA1*, *MALAT1*, *NEAT1*, and *IGFBP3* expression by normal epithelial cells on the aged matrix, all associated with EMT, cell migration, and cancer invasion and progression.^[^ ^33^, ^34^, ^35^ ^]^ Interestingly, a subset of cells (cluster 7) with highly upregulated EMT markers *ROS1*, *ZEB2*, *FN1*, *VCAN*, and *SPARC* are also enriched in the aged breast microenvironment. Hence, here we show, for the first time, that the aged breast ECM alone can predispose normal epithelial cells to an EMT‐like and invasive phenotype. In line with our data, induced metastatic ability of the ovarian (OvCa)^[^ ^56^ ^]^ and prostate (TRAMP‐C2 and Myc‐CaP)^[^ ^57^ ^]^ cancer cells were reported in the aged microenvironment in mice.

LOX is an EMT marker reported to repress E‐CAD.^[^ ^58^ ^]^ However, here we rather show that LOX is involved in the delocalization of E‐CAD from the cell membrane to cytosol. We show that *LOX* mRNA is upregulated in cells on aged matrices compared to young, and when it is knocked down, E‐CAD in cells on the aged matrix is re‐localized to cell membrane. Remarkably, cytokine levels in the aged microenvironment, especially the uPA/uPAR mediators uPA,^[^ ^59^ ^]^ uPAR, SERPINE1,^[^ ^60^, ^61^ ^]^ ANG,^[^ ^62^ ^]^ and OPN,^[^ ^63^, ^64^ ^]^ as well as the IL1 family^[^ ^65^, ^66^ ^]^ and IL8,^[^ ^67^ ^]^ TGF*β* family,^[^ ^68^ ^]^ MMP2,^[^ ^69^, ^70^ ^]^ which largely affect cell behavior, including proliferation, migration, and differentiation, as well as cancer progression,^[^ ^71^ ^]^ are reduced to the young matrix levels after *LOX* knockdown.

Loss of E‐CAD from cell membrane is reported to increase the expression of uPAR,^[^ ^72^ ^]^ supporting our finding. Our results also indicate that *LOX* knockdown leads to decreased expression of aging‐associated proteins that are involved in cancer progression and cell migration and invasion, including the intracellular (galectin 3, CapG, HO1, SNAI, mesothelin, and AFP), surface (E‐CAD, HER, HER2, HGFR, and thrombospondin 1), and extracellular (MMPs and cathepsins) proteins, cytokines (IL8, MIF, IL1*α*, IL4, CCL2) and growth factors (TGF*β*, GDF15, FGF, and angiopoietin 2). However, *LOX* knockdown also leads to increased levels of the proangiogenic factors (VEGF, PDGF‐AA, and GM‐CSF), which may increase the risk of breast cancer progression.

As LOX is a collagen crosslinking enzyme and increases the stiffness of ECM, targeting LOX activity has previously been proposed to prevent metastasis.^[^ ^20^ ^]^ LOX inhibitors such as *β*‐aminopropionitrile (BAPN) and the aminomethylene pyridine based CCT365623 have proven effective in reducing metastasis in mouse models of breast cancer.^[^ ^73^, ^74^ ^]^ However, along with the active form of LOX enzyme, they reduce the LOX propeptide, which plays a role in inhibiting the pro‐oncogenic *β*‐catenin signaling through localizing *β*‐catenin to the cell membrane.^[^ ^75^ ^]^ Therefore, targeting the active form of LOX instead of using non‐specific anti‐LOX drugs that also target the propeptide would be a more effective strategy in preventing breast cancer progression. Results of our study indicate potential benefits for the use of LOX inhibitors alone or with anti‐angiogenic drugs for old patients who are at the early stages of cancer to prevent its invasion.

## 4 Conclusion

These findings indicate that the aged matrix create an invasive microenvironment for the cells. This study is important because it could lead to a better understanding of cell migration and invasion processes in aged tissues, which would lead to improved prognosis and disease outcome, as well as shedding light to cancer initiation and progression processes, since aged microenvironment may harbor components that could lead to cancer initiation or progression, while lacking other components that might prevent it. In the same vein, a young microenvironment could shed light into new ways to prevent cancer. Analysis of how normal cell transcriptome changes in response to the aging ECM could enable identification of target genes that could be useful in preventing cancer initiation and progression, as well as the underlying ECM components responsible for the cell response. This study would usher the role of aging ECM not only in breast cancer, but also in many cancers and other age‐related diseases, and pave the way for the discovery of more efficient treatment strategies.

In future experiments, we will analyze the effect of age on human breast tissues and examine how human epithelial cells will behave on the healthy aged human tissues.

## 5 Experimental Section

### Tissue Harvest and Decellularization/Delipidation

Fourth mammary glands were harvested from 3–6 months (young) or 20–23 months old (aged) C57BL/6 mice according to the IACUC guidelines (protocol number: 18‐05‐4687) with the approval of the University of Notre Dame, which has an approved Assurance of Compliance on file with the National Institutes of Health, Office of Laboratory Animal Welfare. Mice were sacrificed in CO_2_ chambers, and tissues collected and used immediately, or wrapped in aluminum foil, flash frozen in liquid nitrogen, and stored at −80 °C until use.

To section the tissues in cryostat, tissues were thawed at room temperature (RT), blotted on a tissue paper, embedded in optimum cutting temperature (O.C.T.) compound (Tissue‐Tek, Sakura, USA), frozen at −20 °C, and sectioned at 300 μm thickness. Sections were washed with PBS to remove the optimum cutting temperature (O.C.T) compound.

For decellularization, whole tissues or tissue sections were incubated in 0.5% SDS for 4 and 2 days, respectively, at 4 °C, with gentle agitation and SDS change every 12 h. For delipidation, isopropanol was used again for 4 and 2 days, respectively, at 4 °C. Tissues and sections (final dimensions ≈2 mm × 2 mm × 0.3 mm) were washed with PBS and stored at 4 °C until use (within 2 weeks).

DNA content of the matrices was measured using the PicoGreen assay as described previously.^[^ ^76^ ^]^ Briefly, samples (*n* = 3) were frozen at −80 °C, lyophilized, weighed, incubated in pepsin (Sigma, USA) solution (1 mg mL^–1^) for 16 h, and the supernatants were assayed in reference to double stranded DNA standards. Results were normalized to dry weights.

### Microscopy

To analyze collagen structure, the native breast tissues and matrices (*n* = 3, each sample from a different mouse) were imaged with a two‐photon microscope (Olympus, FV1000 MPE) using second harmonic generation (SHG) imaging at 800 nm, and the collagen fiber thickness was measured using Fiji software (NIH, USA). Four to eight fibers in the center of the images were analyzed from each sample to compare fiber thickness and straightness. Fiber straightness was done by drawing a line along the fiber and dividing the end‐to‐end distance to fiber length.

Native tissues were coated with gold/palladium and analyzed with digital filed emission scanning electron microscope (FEI Magellan 400, USA) at 15 kV voltage under high vacuum.

### Nanoindentation Testing

For the mechanical characterization, a nanoindenter (Piuma Chiaro, Optics11, The Netherlands) with a 10 N load cell, and a silicon nitride SNL‐10 cantilever (Bruker, USA) with a spring constant of around 0.261 N m^–1^ were used. Whole tissue or matrix samples (*n* = 5 for each group, each sample from a different mouse) were tested at 5–20 different locations (each point was 2 mm apart at *x*‐ or *y*‐axis) with a loading velocity of 2 mm s^–1^. Young's modulus was determined by a custom developed MATLAB code using Hertz contact model as described previously,^[^ ^16^ ^]^ assuming a Poisson's ratio of 0.5.

### Cytokine Profiling

The relative content of 111 cytokine proteins (Table S1, Supporting Information) was determined using the dot blot based Proteome Profiler Mouse XL Cytokine Array kit (R&D Systems). For cytokine profiling, samples were either used after being homogenized or placed in serum‐free media to release the cytokines and the media was used. Native mouse tissues or decell/delip matrices (*n* = 3 pooled samples, each from a different mouse) were flash frozen in liquid nitrogen and ground using a mortar and pestle. The powders were suspended in protease inhibitor cocktail (Sigma) solution and homogenized using an ultrasonicator. Triton X‐100 was added at 1% to disrupt cells and fatty components. After removal of the cellular debris and fatty components supernatants were pooled. Alternatively, two decellularized matrices/ mouse were combined (*n* = 4 mice) incubated in 250 μL serum‐free media in 24‐well plates at 37 °C for 7 days and the media from the four samples were collected and pooled. Protein quantification was done using the bicinchoninic acid (BCA) rapid gold assay kit (Pierce, Thermo Fisher Scientific). Samples containing equal amounts of proteins were loaded to membranes and the assay was performed following manufacturer's instructions. Membranes were then imaged using a biomolecular imager (ImageQuant LAS4000, GE Healthcare, USA). Relative cytokine content was determined by quantification of the dot intensities using Fiji.

### Mass Spectrometry

Dried breast tissues (*n* = 3 young and 5 aged samples, each from a different mouse) were lysed using a lysis buffer containing 6 m urea and 2 m thiourea at 4 °C for 48–72 h, and proteins were precipitated in acetone at −20 °C. The pellet was dissolved in 8 m urea buffer containing 8 m urea, 75 mm NaCl, 50 mm Tris‐HCl (pH8.2), 1 mm sodium fluoride, 1 mm *β*‐glycerophosphate, 1 mm sodium orthovanadate, 10 mm sodium pyrophosphate, 1 mm phenylmethylsulfonyl fluoride, and protease inhibitors, followed by sonication and centrifugation to dissolve the tissue and remove pellets. Samples containing 100 μg protein (quantified with BCA assay) were treated with 5 mm dithiothreitol for 25 min at 56 °C, and 14 mm iodoacetamide for 30 min at RT in dark. The protein mixture was then diluted with 25 mm Tris‐HCl (pH 8.2) to achieve a final urea concentration of 1.8 m. Trypsin (Sigma‐Aldrich) (final concentration: 0.005%) and CaCl_2_ (final molarity: 1 mm) were added and the protein solution was incubated overnight at 37 °C. Trifluoroacetic acid (final concentration: 0.4%) was added to stop digestion reaction. Samples were cleaned with ZipTip pipette tips and re‐suspended in MS loading buffer (1% HPLC grade acetonitrile [ACN], 0.1% formic acid [FA] in HPLC grade water).

The liquid chromatography electrospray ionization tandem mass spectrometry (LC‐ESI‐MS/MS) was performed on a Q‐Exactive mass spectrometer (Thermo Fisher Scientific) equipped with a nanoelectrospray ion source operating at a source voltage of 1.8 kV, and an ion transfer tube temperature of 280 °C and coupled with a nanoACQUITY Ultra Performance LC (UPLC) system (Waters Corporation). Peptides were dissolved in a buffer containing 0.1% FA and 3% CAN, and loaded onto a C18 reverse phase column (100 μm × 100 mm, 1.7 μm particle size, BEH130) (Waters Corporation). Peptide separation was carried out with a 73‐min linear gradient from 3% to 40% FA in ACN. The full MS scans were acquired in the Orbitrap mass analyzer with an *m*/*z* range of 350–1800, at the mass resolution of 70 000 at *m*/*z* = 200. Automatic gain control (AGC) target value was set to 1 × 10^6^, with a maximum fill time of 250 ms. For the MS/MS method, the top 12 most intense parent ions were selected with an isolation window of 2.0 *m*/*z* and fragmented under a normalized collision energy of 30%, with the AGC target value of 1 × 10^6^ and the maximum fill time of 120 ms. The parent ions with unassigned charges or a charge state of *z* = 1 were excluded from fragmentation. The intensity threshold for selection was set to 8.3 × 10^4^. The fragmentation was performed in an HCD collision cell with a mass resolution of 35 000 at *m*/*z* = 200, and a dynamic exclusion period of 20 s after 1 repeat count. Samples were run in triplicates at a 1000 nL min^–1^ flow rate.

All raw files acquired with the Q‐Exactive were searched with the Global Proteome Machine. The peptide false discovery rate (FDR) was determined by searching against the corresponding reverse database. The search was performed with precursor peptide mass tolerance of 10 ppm and fragment ion mass tolerance of 0.02 Da. Carbamidomethylation of cysteine was set as a fixed modification, while oxidation of methionine was set as a variable modification (FDR = 0.01).

Only the most highly expressed 11–13 structural proteins are presented, either as the protein levels in aged tissue relative to those in the young^[^ ^77^ ^]^ or as the protein counts in a tissue normalized to the total counts of the structural proteins in that tissue.^[^ ^78^, ^79^ ^]^

### Western Blotting

Native tissues were placed in liquid nitrogen, pulverized, and incubated in RIPA buffer for 30 min on ice, and centrifuged at 10 000 g for 10 min. Protein quantification of the supernatant was done using the BCA assay. Proteins (10 μg) were loaded into polyacrylamide gels (12%) and the samples run at 200 V for 45 min. The proteins were transferred to nitrocellulose membranes at 100 V for 1 h, blocked in blocking buffer (Thermo Fisher Scientific), and incubated overnight in rabbit anti‐mouse Col1 (Abcam) and rabbit anti‐beta actin (Abcam) at 1:1000 dilution, followed by a 1 h incubation in the HRP‐labelled goat anti‐rabbit IgG (Abcam) at 1:2000 dilution. Next, the membranes were incubated in chemiluminescent substrate (Clarity ECL, Bio‐Rad) for 5 min and imaged under a ChemiDoc‐It2 (UVP, USA).

### Immunohistochemistry and Histology

Native tissues were fixed in 4% paraformaldehyde (PFA) solution, embedded in paraffin, sectioned at 6 μm thickness, attached to positively charged slides, and deparaffinized and rehydrated.

For collagen I staining, sections were incubated for 5 min in 0.3% Triton X‐100, for 45 min in 5% goat serum, overnight at 4 °C in rabbit anti‐mouse collagen I antibody (Abcam, USA, dilution: 1:100), and for 1 h at RT in goat anti‐rabbit IgG (Abcam, dilution: 1:400). The samples were covered with Prolong Gold antifade reagent with DAPI (Cell Signaling Technology, USA) prior to imaging under an inverted fluorescence microscope (Zeiss Axio Observer.Z1).

Hematoxylin and eosin (H&E) and Masson's trichrome staining were performed at Notre Dame Integrated Imaging Facility (University of Notre Dame) and the sections imaged using a bright field microscope (Nikon, Eclipse ME600, USA).

### Cell Seeding and Culture

KTB21 human mammary basal epithelial cell line (transformed with human telomerase gene using the vector pLXSN‐hTERT) was previously established in Dr. Harikrishna Nakshatri's lab from a 40 year‐old patient.^[^ ^28^ ^]^ KTB21 cells were cultured in epithelial cell growth medium (DMEM [low glucose]:Ham's F12 [1:3] medium supplemented with 5% FBS [Thermo Fisher Scientific], 0.4 μL mL^–1^ hydrocortisone [Sigma], 1% penicillin/streptomycin [Corning], 5 μg mL^–1^ insulin [Sigma], 10 ng mL^–1^ EGF [Millipore], 6 mg mL^–1^ Adenine [Sigma], and 10 mm ROCK inhibitor [Y‐27632] [Enzo Life Sciences]). Decell/delip matrices were sterilized in a solution containing 4% ethanol and 0.15% peracetic acid in PBS, washed with PBS and then with media, and placed in 96‐well culture plates. KTB21 cells were detached from culture plates using trypsin‐EDTA (0.25%), reconstituted in epithelial cell growth medium, and seeded on matrices.

For KTB21 cell spheroid experiments, matrices (*n* = 3) were coated with 20 μL Matrigel (growth factor reduced, phenol red‐free, and LDEV‐free) (Corning, USA) and incubated for 10 min at 37 °C, to induce KTB21 acini‐like spheroid formation, since no spheroid formation was observed on the Matrigel‐free matrices (Figure S10, Supporting Information). Matrices were seeded with cells and incubated in a CO_2_ incubator, with media change every 2–3 days. Spheroid formation and stability were monitored for 15 days.

For cell migration and invasion experiments, KTB21 and the GFP‐reporting MDA‐MB‐231.BR cells (a gift from Dr. Patricia Steeg at NCI) were seeded on the Matrigel‐free matrices, and incubated in epithelial and cancer cell growth medium (DMEM [high glucose] supplemented with 10% FBS and 1% penicillin/streptomycin), respectively, with media change every 2–3 days.

### LOX siRNA Treatment and Knockdown Confirmation

For knockdown experiments, scramble (Cy3‐labeled negative control) (Thermo Fisher Scientific), and LOX (Thermo Fisher Scientific) siRNAs were applied for 48 h. Briefly, siRNAs (80 nm in serum free medium) and the Lipofectamine RNAiMax transfection reagent (Thermo Fisher Scientific) (12 μg mL^–1^ in serum free medium) were mixed at 1:1 volume ratios, incubated for 5 min at RT, and applied on cells on culture plates or on matrices after washing the cells with PBS. To verify mRNA and protein knock down, quantitative reverse transcriptase‐polymerase chain reaction (qRT‐PCR) and western blotting were performed for cells on culture plates after incubating cells for 48 h in the siRNA solutions.

For qRT‐PCR, RNAs were collected using the RNA isolation kit (RNeasy, Qiagen), and cDNAs were synthesized using the iScript cDNA Synthesis Kit (Bio‐Rad). Quantitative PCR was done using the iTaq SYBR Green Supermix (Bio‐Rad) with certified human GAPDH and LOX primers (Bio‐Rad). The reaction was run in CFX Connect 96 Real Time PCR system (Bio‐Rad). The ΔΔCq method was applied to quantify the relative expression of genes, and GAPDH was used as the housekeeping gene.

For KTB21 cell spheroid experiments, siRNAs were applied for 48 h between days 8–10 after cell seeding on matrices. For KTB21 cell invasion experiments, siRNAs were applied two times for 48 h each (between days 8–10 and days 15–17 of cell seeding on the matrices). For MDA‐MB‐231 cell invasion experiments, siRNAs were applied for 48 h, between days 5–7. For all cell migration experiments, siRNAs were applied on cells on culture plates for 48 h just before cells were seeded on matrices.

### Spheroid Formation, Live/Dead Staining, and Immunostaining

Viability of KTB21 cells in spheroids was determined using the live/dead cell viability assay (Thermo Fisher Scientific). Briefly, matrices (*n* = 3) at day 14 of culture were incubated in a solution of 2 μ m calcein‐AM (live cells, green) and 4 μ m ethidium homodimer‐1 (EthD‐1) (dead cells, red) at 37 °C for 30 min, and cells imaged using a fluorescence microscope. Cell viability was calculated as the percentage of live cells (green) in the total cell count (green and red). Spheroid morphology (circularity and aspect ratio) was analyzed from the live/dead images using Fiji. Spheroids on matrices (*n* = 2) were also imaged using SHG at day 15 of culture.

Untreated (no siRNA) samples (*n* = 3) were stained for E‐CAD or co‐stained for MMP2 and COL1. LOX siRNA‐treated samples (*n* = 4) were double stained for E‐CAD and MMP2 or for LOX and MMP9. Briefly, matrices at day 15 of culture were fixed with 4% PFA and permeabilized with 0.3% Triton X‐100, and then incubated for 45 min in 5% goat serum, overnight at 4 °C in mouse anti‐human E‐CAD (Abcam), mouse anti‐human COL1 (Abcam), rabbit anti‐human MMP2 (CusaBio), rabbit anti‐human MMP9 (Abcam), and mouse anti‐human LOX (LSBio) monoclonal antibodies with 1:100 dilutions, and then for 1 h in Alexa fluor 488‐labelled goat anti‐mouse IgG (Abcam), and Alexa fluor 647‐labelled goat anti‐rabbit IgG (Abcam) with 1:400 dilutions. The samples were stained with 0.5 μg mL^–1^ DAPI (Sigma) and imaged with a two‐photon confocal microscope or inverted fluorescence microscope. SHG imaging was done along with COL1 to verify that the collagen was produced by the cells.

For western blotting, cells in tissue culture plates or on matrices (both native and siRNA treated) were lysed by incubating in RIPA buffer for 30 min on ice. Protein quantification was done using the BCA assay. Equal amounts of proteins (15 μg) were loaded into polyacrylamide gels (8–12%) and the samples run at 125 V for 2 h or 200 V for 45 min. The proteins were transferred to nitrocellulose membranes at 100 V for 1 h or 200 V for 40 min, blocked in BSA solution (5%) or EveryBlot blocking solution (Bio‐Rad), and incubated overnight in mouse anti‐human LOX (LSBio) or rabbit anti‐human LOX (Abcam), mouse or rabbit anti‐human E‐CAD (Abcam), and rabbit anti‐human beta actin (Cell Signaling Technology) at 1:500 dilutions, followed by a 1 h incubation in the HRP‐labelled horse anti‐mouse IgG (Cell Signaling Technology), and goat anti‐rabbit IgG (Cell Signaling Technology) at 1:1000 dilutions. Next, the membranes were incubated in chemiluminescent substrate (SuperSignal West Pico PLUS, Thermo Fisher Scientific or Clarity, Bio‐Rad) for 5 min and imaged.

### Migration and Invasion Assays

For migration assays, KTB21 and GFP‐reporting MDA‐MB‐231 cells were seeded on culture plates and incubated until around 70% confluence. To track KTB21 cells under microscope, they were stained with 10 μ m CellTracker Green 5‐chloromethylfluorescein diacetate (CMFDA) (Thermo Fisher Scientific) for 30 min. Cells were seeded on the Matrigel‐free matrices at 2 × 10^6^ cells per milliliter (around 5 × 10^4^ cells/matrix) density. After 24 h, cells were imaged with fluorescence microscope at 15 min intervals for 4–20 h. Time‐lapse images were analyzed using the MTrackJ plugin in Fiji to track cell movements, and converted to .mpeg video files. Only the clumped cells were eliminated, because these cells are difficult to track and analyze. Cell trajectories were plotted and motility was calculated based on the distance travelled by the cells in a specific time period. All the migration experiments were performed as two independent experiments.

For invasion assays, cells were seeded on Matrigel‐free matrices at 4 × 10^6^ cells per milliliter (around 10^5^ cells/matrix) density, and pre‐cultured until they populated the matrices. KTB21 cell‐seeded matrices were transferred at day 7 to transwell inserts (pore size: 8 μm) (Corning) containing epithelial growth medium (5% FBS), and incubated against 10% FBS gradient until day 21. Alternatively, to test the effect of cytokines in the matrices on cell invasion, KTB21 cells (10^4^ cells/well) were seeded in the transwell and the cell‐free matrices were placed in the bottom chambers as incentives for invasion. Cells were incubated for 14 days. MDA‐MB‐231 cell‐seeded matrices were transferred at day 7 to transwell inserts containing serum‐free cancer cell growth medium, and incubated against 10% FBS gradient in the bottom well until day 11. At the end of invasion tests, live cells in the bottom chambers were counted manually to assess cell invasion. All invasion experiments were performed twice as two independent experiments.

### Single‐Cell RNA‐Sequencing

KTB21 cells were seeded on six matrices per age group (three pieces per mouse, two different mice per group), incubated on the Matrigel‐coated matrices for 20 days. Samples were treated with trypsin‐EDTA (0.25%) to collect the cells. Cells from the three matrices obtained from the same mouse were pooled and treated as one biological sample. Preparation for scRNA‐seq was performed as described elsewhere.^[^ ^80^ ^]^ Briefly, cells were resuspended in PBS containing 2% BSA and 0.02% Tween 20 at 1 million cells per milliliter density, and blocked by incubating in the Human TruStain FcX blocking solution (Biolegend, 422301) for 20 min on ice. Next, cells were incubated in human hashtag (HTO) antibodies (Biolegend, dilution: 1:300) for 25 min on ice. Finally, cells were washed four times in a series of buffers (first wash: 2% BSA, 0.02% Tween 20 in PBS; second wash: 2 mm EDTA, 2% BSA, 0.02% Tween 20 in PBS; third wash: 1 mm EDTA, 2% BSA, 0.02% Tween 20 in PBS; fourth wash: 0.1 mm EDTA, 1% BSA, and 0.02% Tween 20 in PBS). Cells were pooled prior to the final wash, counted, and resuspended at 1500 cells/μL.

10× Genomics Chromium was used for single cell capture, cDNA libraries were prepared according to the standard CITE‐seq and 10× Genomics standard protocols. The resulting HTO‐derived and mRNA‐derived cDNA libraries were pooled and sequenced. 26 bp of cell barcode and UMI sequences and 91 bp RNA reads were generated with Illumina NovaSeq 6000. The raw base sequence calls generated from the sequencer were demultiplexed into sample‐specific mRNA, ADT, and HTO FASTQ files with bcl2fastq through CellRanger 3.1.0. Raw FASTQ files were processed using Cellranger 3.1.0.

Data analysis was performed in R (v 3.6.2) using the Seurat package (v 3.1.2)^[^ ^81^ ^]^ for data normalization, dimension reduction, clustering, and differential gene expression analysis. Samples were demultiplexed by HTO expression with a positive quantile of greater than 0.99. For quality control, cells with greater than 1000 mRNA transcripts, and less than 20% mitochondrial genes were kept for analysis. To ensure even comparisons between cells cultured on young and aged matrices, each biological sample was randomly made subset to include 1300 cells. Dimension reduction and clustering was performed using standard parameters for the combined young and aged matrix datasets, followed by determination of the number of cells from each experimental condition present in each cluster. ssGSEA analysis was performed using GSVA (v 1.34.0) with all C2 gene sets from the Molecular Signatures Database. Gene ontology analysis was performed using the EnrichR website, as previously reported.^[^ ^82^ ^]^ The biological processes upregulated in cells on the aged matrix were plotted.

### Cytokine and Oncology Arrays

For cytokine analysis, the dot blot‐based Proteome profiler human XL cytokine array kit, which detects 105 cytokine proteins (Table S4, Supporting Information), and human XL oncology array kit (R&D Systems), which detects 84 human cancer‐related proteins (Table S5, Supporting Information), were used as described above. Briefly, cell lysates of the KTB21 cell‐seeded matrices (spheroid experiment) were collected at day 15. After protein quantification with the BCA assay, equal amounts of proteins were loaded onto membranes. Relative cytokine content was determined after quantification of the dot intensity using Fiji.

### GTEx and TCGA Analyses

Correlation between LOX and E‐CAD, MMP2, and MMP9 for the breast tissue (GTEx) and normal and tumorous breast tissues (TCGA, invasive breast carcinoma) data sets was done using the GEPIA database (http://gepia.cancer‐pku.cn/).^[^ ^83^ ^]^ Survival of breast cancer patients in TCGA data set based on their LOX expression, cancer type, and menopause status was generated in the UALCAN cancer database (http://ualcan.path.uab.edu).^[^ ^84^ ^]^

### Statistical Analysis

Data were analyzed for statistical significance using GraphPad Prism 6 or R software. Two‐tailed unpaired student's *t*‐test was used to compare the difference between two groups and one‐way ANOVA followed by Tukey's HSD correction were performed to compare the differences between multiple groups. Two‐way ANOVA was applied to test the effect of two independent variables (age and siRNA treatment) on a dependent variable (motility and invading cell number), and whether there is an interaction between the two independent variables. For scRNA‐seq, all statistical tests were performed using Seurat version 4.0.1, which applies Wilcoxon Rank Sum test by default. Samples were eliminated only when they were degraded during cell culture or if they were outliers. Outliers were identified using the ROUT method with *Q* = 1%. To test differences in variances, F test was done after student's *t*‐test and Brown–Forsythe test after ANOVA. Welch's correction was applied after Student's *t*‐test when variances were unequal. *P* was adjusted for multiple comparisons. Data are presented as the mean ± standard deviation (SD).

## Conflict of Interest

The authors declare no conflict of interest.

## Supporting information

Supporting Information

Supplemental Movie 1

Supplemental Movie 2

Supplemental Movie 3

Supplemental Movie 4

Supplemental Movie 5

Supplemental Movie 6

Supplemental Movie 7

Supplemental Movie 8

Supplemental Movie 9

Supplemental Movie 10

Supplemental Movie 11

## Data Availability

The data that support the findings of this study are available from the corresponding author upon reasonable request.
