# Supplementary material for: Aged Breast Extracellular Matrix Drives Mammary Epithelial Cells to an Invasive and Cancer‐Like Phenotype
Source: Adv Sci (Weinh). 2021 Oct 7;8(22):2100128. doi: 10.1002/advs.202100128 (PMC8596116; doi:10.1002/advs.202100128)
Supplement: Supplementary file 1 — Supporting Information [file ADVS-8-2100128-s012.pdf]

© 2021 Wiley-VCH GmbH

ADVANCED SCIENCE
Open Access

Supporting Information

for *Adv. Sci.,* DOI: 10.1002/advs202100128

Aged Breast Extracellular Matrix Drives Mammary Epithelial Cells to an Invasive and Cancer-Like Phenotype

*Gokhan Bahcecioglu, Xiaoshan Yue, Erin Howe, Ian Guldner, M. Sharon Stack, Harikrishna Nakshatri, Siyuan Zhang, Pinar Zorlutuna**

Supplementary Information for
Aged Breast Extracellular Matrix Drives Mammary Epithelial Cells to an Invasive and Cancer-Like Phenotype

Gokhan Bahcecioglu, Xiaoshan Yue, Erin Howe, Ian Guldner, M. Sharon Stack, Harikrishna Nakshatri, Siyuan Zhang, Pinar Zorlutuna

Pinar Zorlutuna
Email: pzorlutu@nd.edu

Other supplementary materials for this manuscript include the following:

Movies S1 to S11

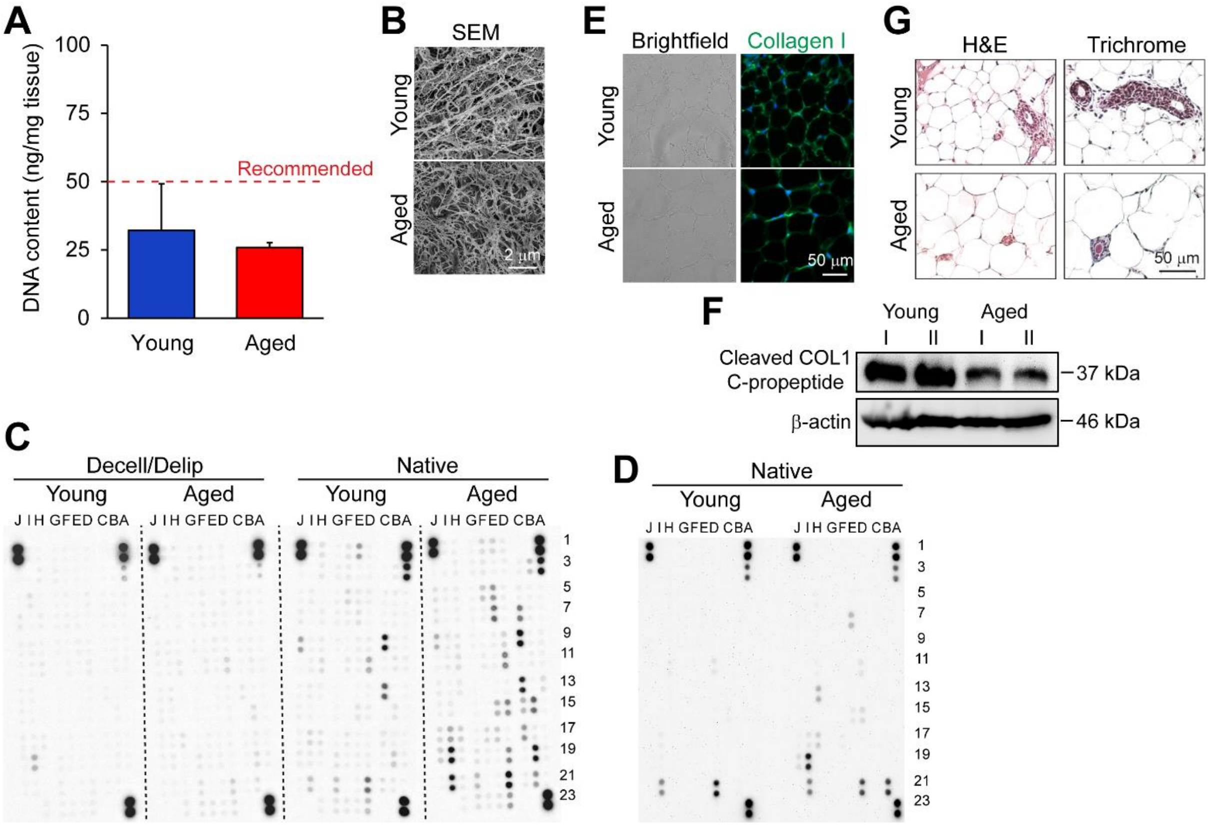

Figure S1. Aging microenvironment alters the structure, biochemistry, and histology of the breast tissue. (A) Picogreen quantification of the double stranded DNA content (ng DNA per mg of dry tissue weight) in the decellularized/delipidized matrices. Dashed line: maximum allowed level for a successful decellularization. n=3 samples/group (each sample from a different mouse). (B) Scanning electron microscopy images of the native breast tissues. (C, D) Dot blots showing the cytokines (C) in mouse breast tissues (n=3 pooled samples/group, each sample from a different mouse), and those (D) released to serum-free culture media after incubation at 37 ^{o}C for 7 days (n=4 pooled samples, each sample from a different mouse, each pooled sample contained two matrix pieces/mouse). Also see Table S1. A1 and A2, A23 and A24, and J1 and J2 are reference spots, and J23, and J24 are negative controls. (E) Bright field (left) and fluorescence (right) microscopy images after collagen staining of the native mouse breast tissues. Green: collagen 1 (Alexa fluor 488), and blue: nuclei (DAPI). (F) Western blots showing the amount of collagen I in the tissues. n=2 biological replicates. Results repeated in two independent experiments. (G) Bright field microscopy images showing histology staining. Left: hematoxylin and eosin (H&E) staining, and right: Masson's trichrome staining.

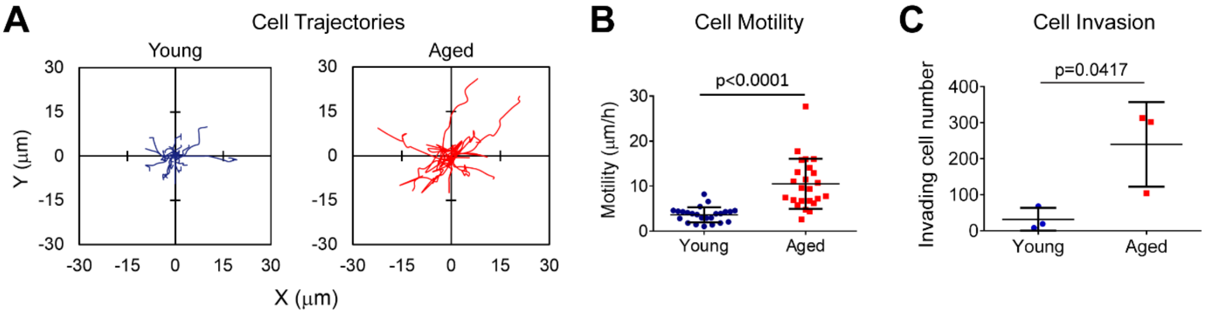

Figure S2. Motility and invasiveness of the MDA-MB-231 cells increase on aged matrix. (A, B) Migration of cells on the matrices. Time-lapse duration: 3 h. (A) Cell trajectories, and (B) motility. Also see Movies S3 and S4 (Supporting Information). (C) Cell invasion through transwell inserts. Cells were seeded on the matrices and pre-incubated for 7 days, and then the matrices were incubated in transwell inserts for 4 days against a 10% FBS gradient. n=3 matrices.

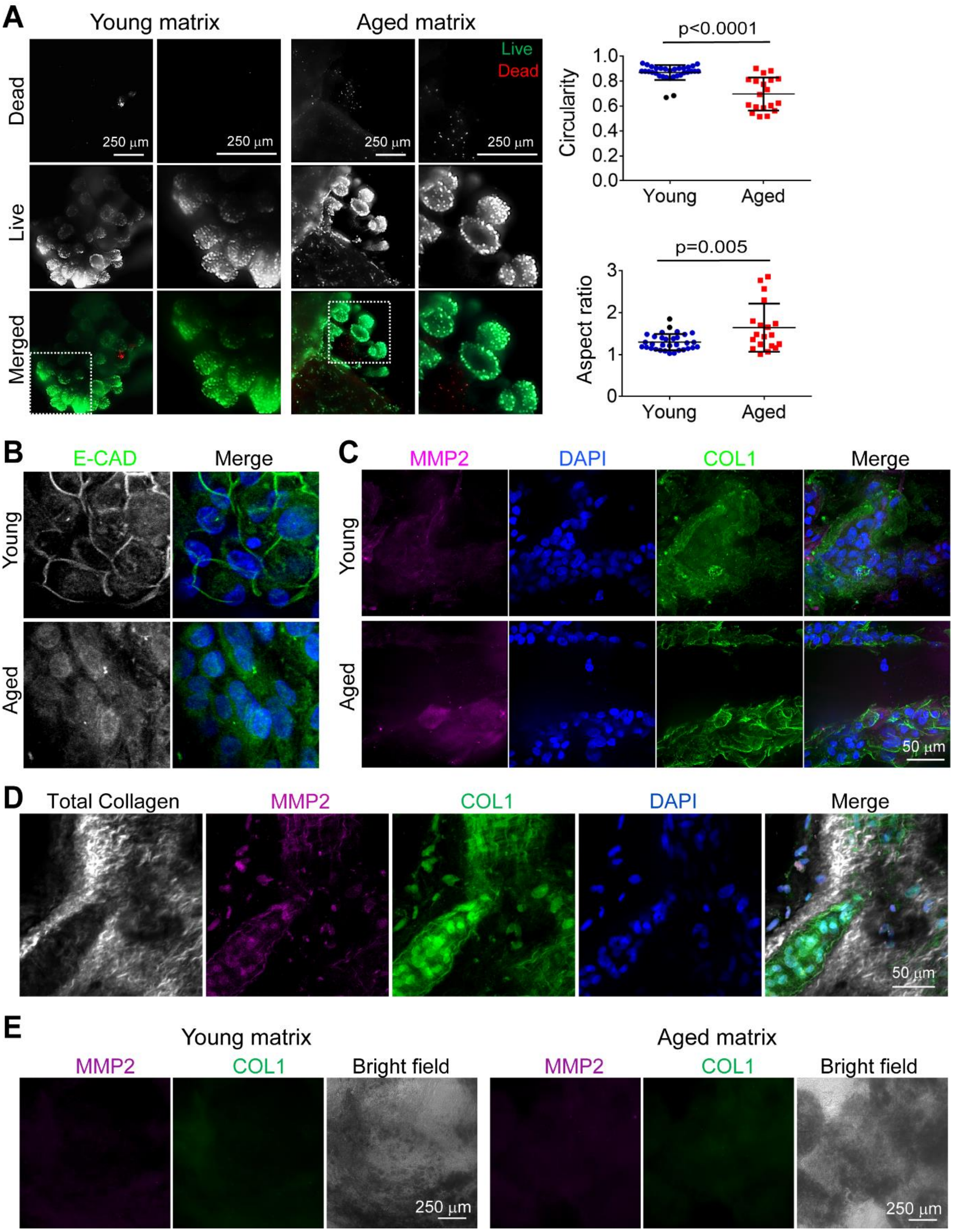
A
Young matrix
Aged matrix
Live
Dead
Dead
Live
Merged
250 μm
p<0.0001
Circularity
1.0
0.8
0.6
0.4
0.2
0.0
Young
Aged
p=0.005
Aspect ratio
3
2
1
0
Young
Aged
B
E-CAD
Merge
Young
Aged
C
MMP2
DAPI
COL1
Merge
Young
Aged
50 μm
D
Total Collagen
MMP2
COL1
DAPI
Merge
50 μm
E
Young matrix
Aged matrix
MMP2
COL1
Bright field
250 μm

Figure S3. Live dead, and E-CAD, MMP2 and COL1 staining of human KTB21 cells on the mouse breast matrices. (A) Live-dead stained KTB21 spheroids on matrices (day 14). Left: fluorescence microscopy images showing cell viability in spheroids. Dashed squares: magnified regions. Green: live (calcein-AM), red: dead (ethidium homodimer-1). n=3. Right: quantification of the circularity and aspect ratio of the spheroids. Outliers are shown as dark color dots. (B) E-CAD staining showing the localization in cells. Green: human E-cadherin (Alexa fluor 488). (C, D) Human MMP2 and COL1 staining on cell-seeded mouse breast matrices. (C) Confocal laser scanning microscopy images showing MMP2 and COL1 staining on matrices. (D) Confocal and SHG imaging combined to distinguish the native type I collagen in the aged matrices and the collagen deposited by the cells. (E) COL1 and MMP2 staining of the cell-free decellularized matrices. Magenta: MMP2, green: COL1. Blue: nuclei (DAPI).

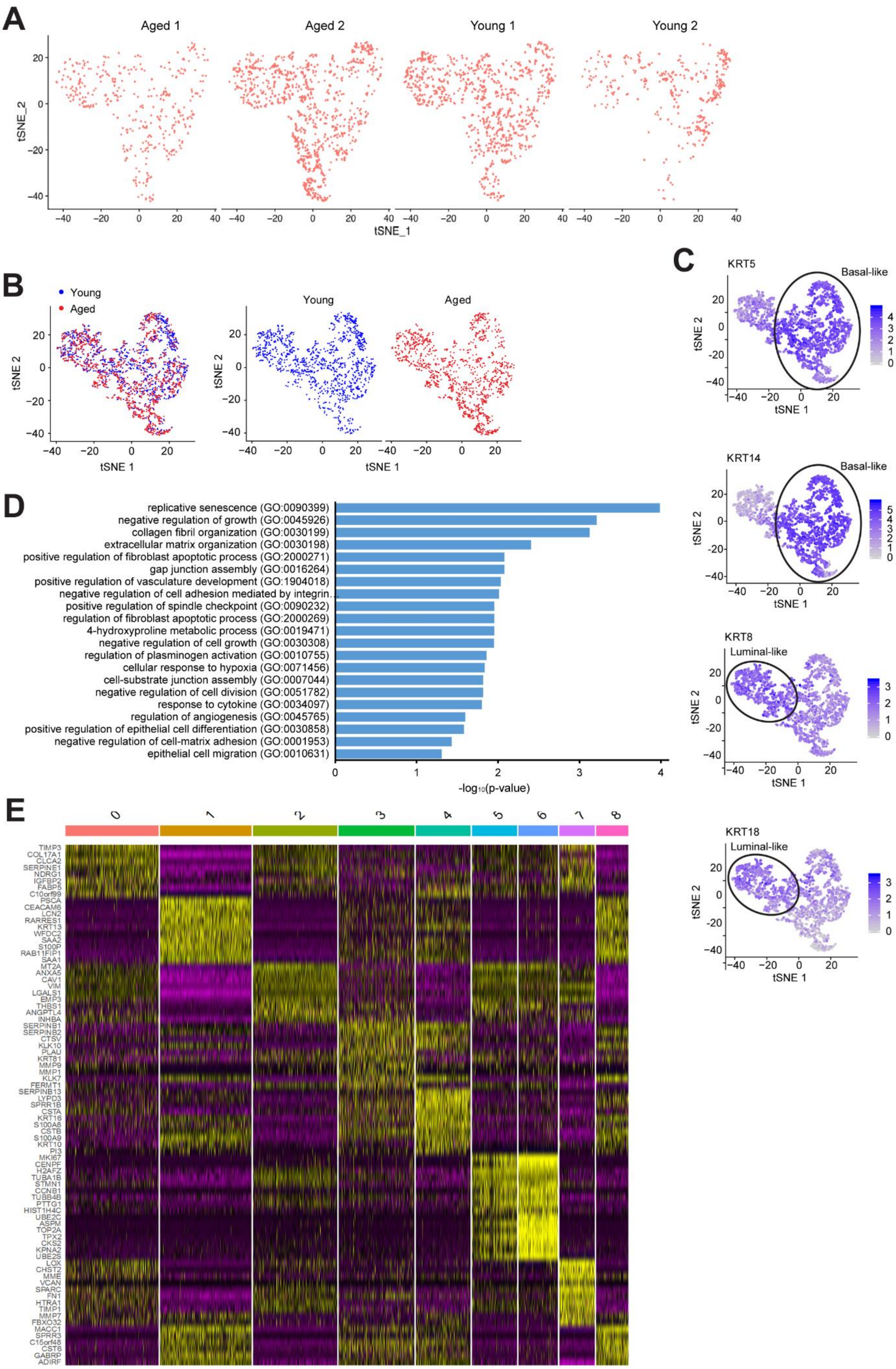

A
Aged 1
Aged 2
Young 1
Young 2
tSNE_1
tSNE_2
B
Young
Aged
tSNE 1
tSNE 2
C
KRT5
Basal-like
KRT14
Basal-like
KRT8
Luminal-like
KRT18
Luminal-like
D
replicative senescence (GO:0090399)
negative regulation of growth (GO:0045926)
collagen fibril organization (GO:0030199)
extracellular matrix organization (GO:0030198)
positive regulation of fibroblast apoptotic process (GO:2000271)
gap junction assembly (GO:0016264)
positive regulation of vasculature development (GO:1904018)
negative regulation of cell adhesion mediated by integrin...
positive regulation of spindle checkpoint (GO:0090232)
regulation of fibroblast apoptotic process (GO:2000269)
4-hydroxyproline metabolic process (GO:0019471)
negative regulation of cell growth (GO:0030308)
regulation of plasminogen activation (GO:0010755)
cellular response to hypoxia (GO:0071456)
cell-substrate junction assembly (GO:0007044)
negative regulation of cell division (GO:0051782)
response to cytokine (GO:0034097)
regulation of angiogenesis (GO:0045765)
positive regulation of epithelial cell differentiation (GO:0030858)
negative regulation of cell-matrix adhesion (GO:0001953)
epithelial cell migration (GO:0010631)
-log10(p-value)
E

Figure S4. KTB21 basal epithelial cells retain their basal phenotype. (A) t-SNE showing the distribution of cells in two biological samples of aged and young matrices. (B) t-SNE showing differential gene expression of all cells on the young and aged matrices. (C) t-SNE showing the expression of basal epithelial markers, KRT5 and KRT14, and luminal epithelial markers, KRT8 and KRT18. (D) Gene ontology analysis showing the biological processes enriched in cells on aged matrices. (E) Heat map showing the top 10 genes that identify each cluster of cells on the matrices.

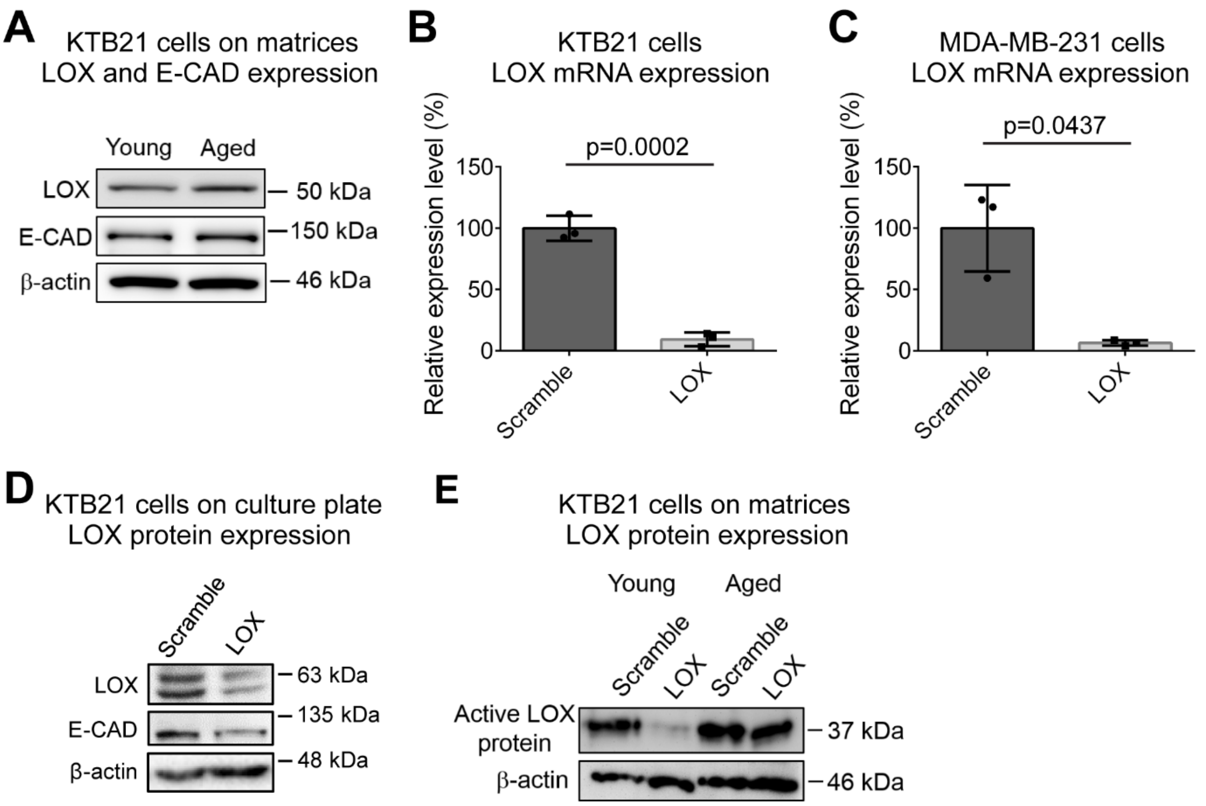

Figure S5. LOX knockdown verification. (A) Western blots showing LOX protein expression on matrices. n=4 pooled samples/group. (B, C) qRT-PCR results showing the *LOX* mRNA expression after scramble and *LOX* siRNA treatment. (B) KTB21 cells, and (C) MDA-MB-231 cells. n=4 samples/group. (D) Western blots showing LOX and E-cadherin protein expression after scramble and *LOX* siRNA treatment of cells on culture plate. n=3 pooled samples. (E) Western blots showing LOX protein expression after scramble and *LOX* siRNA treatment of cells on young and aged matrices. n=3 pooled samples/group. β-actin was used as the reference protein. Student's t tests were applied to test the difference between scramble and *LOX* siRNA treatment groups in (B) and (C).

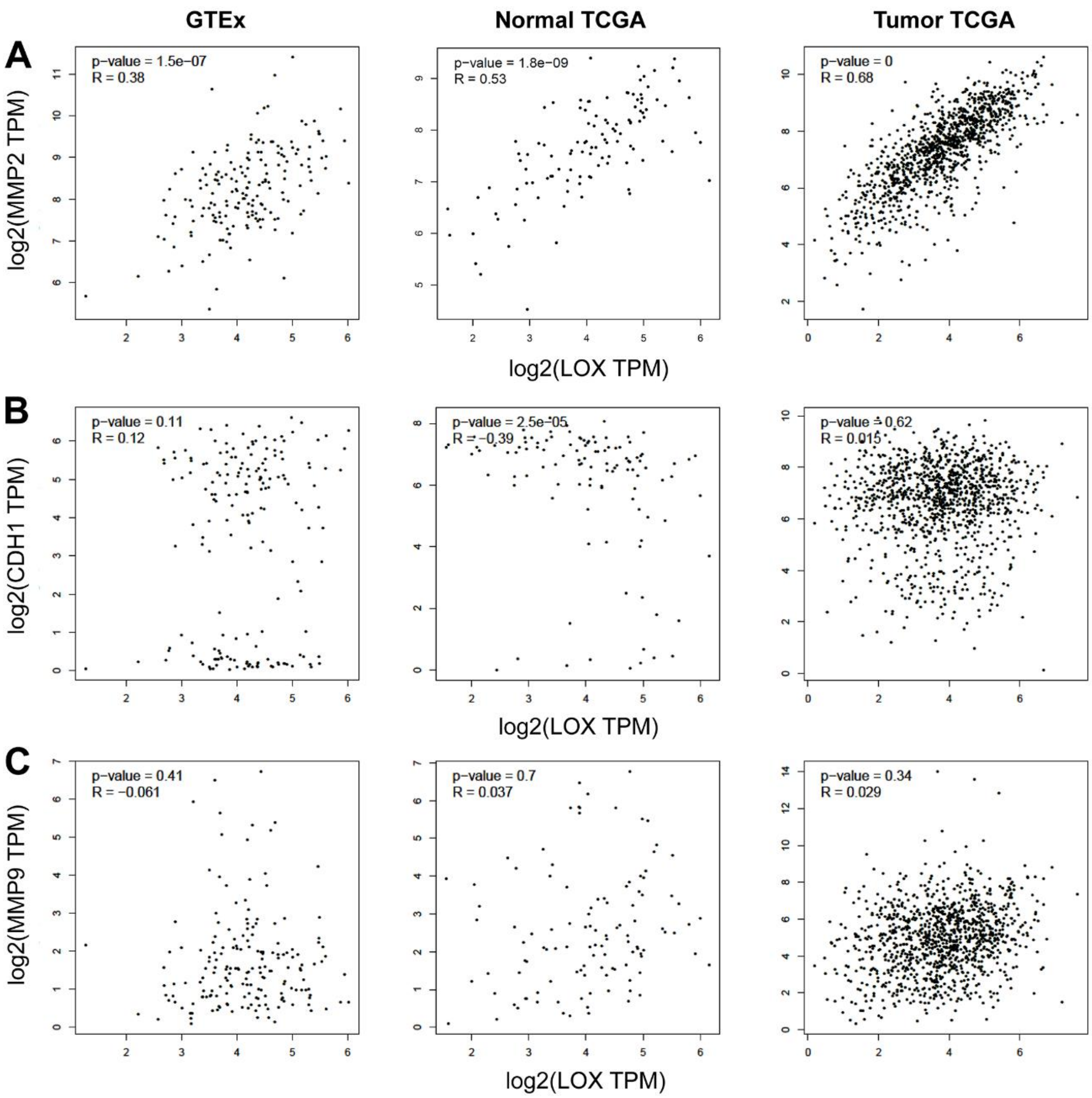

Figure S6. Correlation of LOX and E-cadherin (CDH1), MMP2, and MMP9 mRNA expression in breast tissue in Genotype-Tissue Expression (GTEx) and The Cancer Genome Atlas (TCGA) data sets. Correlation between (A) LOX and MMP2, (B) LOX and E-CAD, and (C) LOX and MMP9. Graphs were generated using the GEPIA website (http://gepia.cancer-pku.cn/).

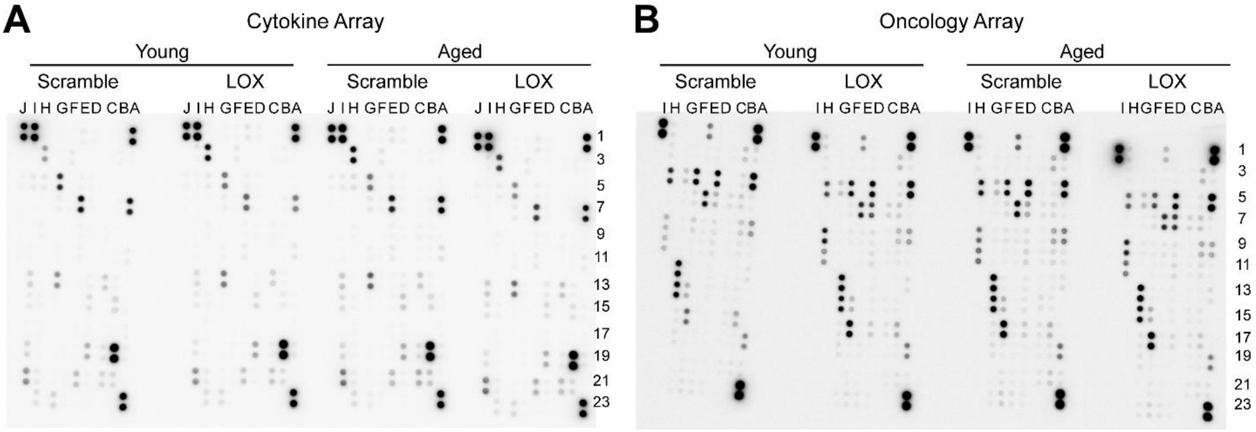

Figure S7. Dot blots of the KTB21 cell lysates showing the effect of LOX knockdown on cytokine production on matrices. (A) Cytokine array, and (B) oncology array results of the KTB21 cells cultured for 15 days on matrices. siRNAs were applied between days 8-10 for 48 h. For (A) also see Table S2. A1 and A2, A23 and A24, and J1 and J2 are reference spots, and J23, and J24 are negative controls. For (B) also see Table S3. A1 and A2, A23 and A24, and I1 and I2 are reference spots, and I23, and I24 are negative controls.

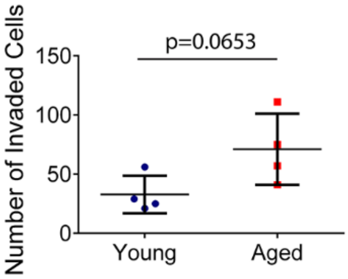

Figure S8. Invasion of KTB21 cells on matrices through transwell inserts. Cells were seeded on matrices and pre-incubated for 7 days in culture plates before they were transferred into transwell inserts. A 10% FBS gradient was used as an incentive for cell invasion. Invaded cell numbers are shown after a 14-day incubation in transwell inserts.

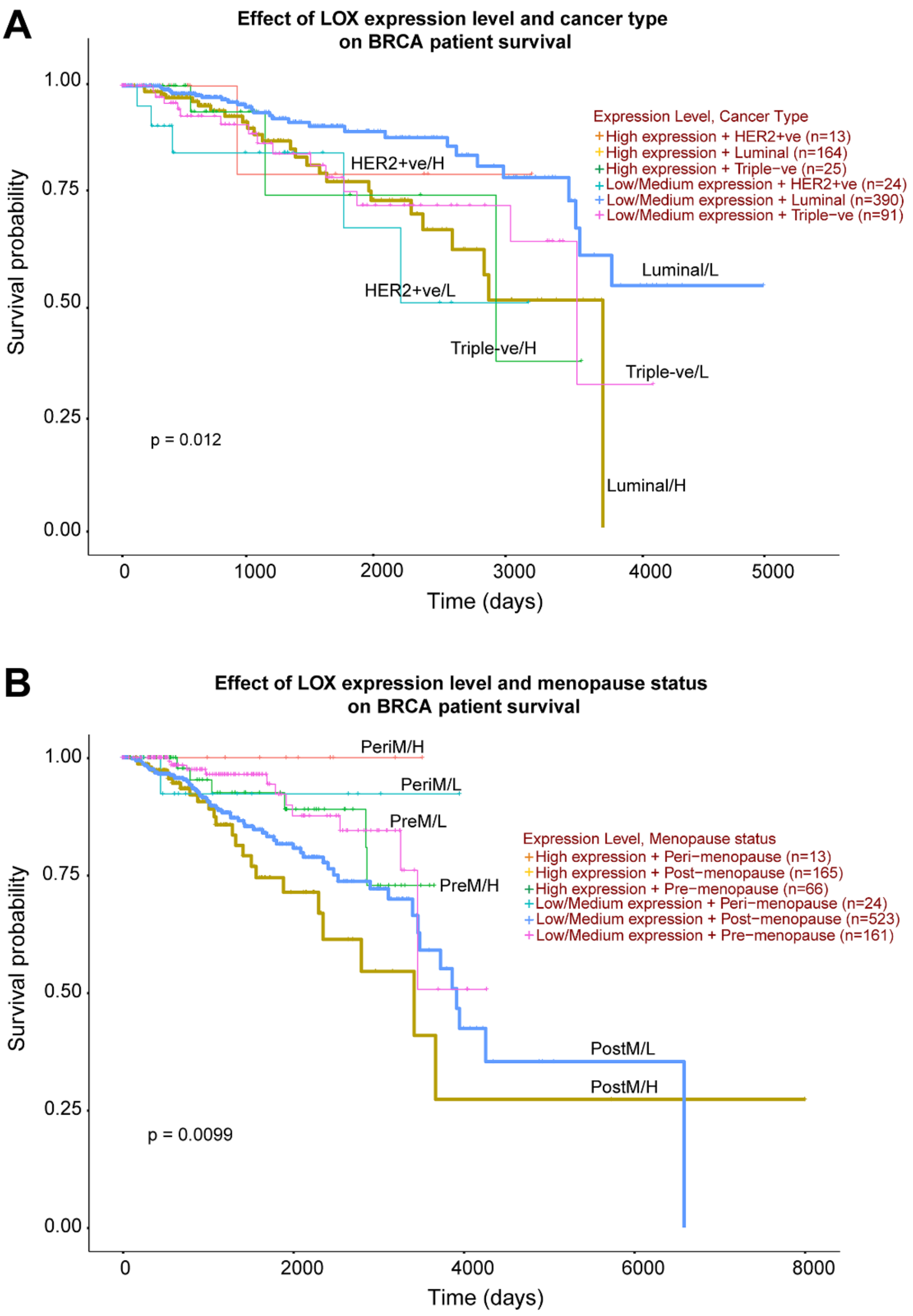

Figure S9. Effects of LOX expression level and cancer type or menopause status on BRCA patient survival in TCGA data set invasive breast cancer cohort. Kaplan-Meier plots showing the survival probability with respect to (A) LOX expression level and cancer type, and (B) LOX expression level and menopause status on breast cancer patient survival. Graphs were generated using the UALCAN website (http://ualcan.path.uab.edu/).

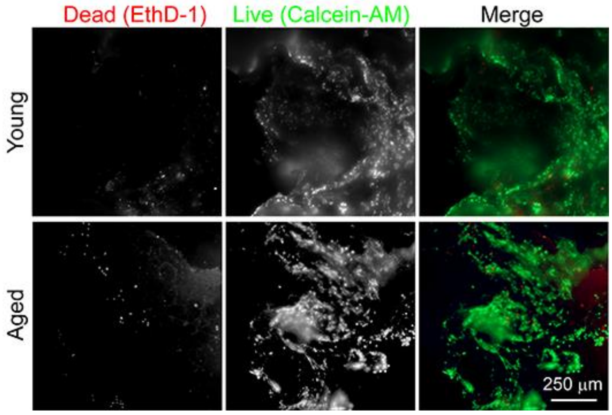

Figure S10. KTB21 cells cannot form spheroids on the decellularized matrices. Fluorescence microscopy images of live/dead stained KTB21 cells at day 14 of incubation on the decell/delip matrices. Red: dead cells (ethidium homodimer-1 (EthD-1)), and green: live cells (calcein-AM).

Table S1. Mouse cytokines tested in dot blot assay

Spot Location	Protein	Spot Location	Protein	Spot Location	Protein
**A1, A2**	Reference Spots	**D15, D16**	Cd26	**G21, G22**	Il-33
**A3, A4**	Adiponectin	**D17, D18**	Egf	**G23, G24**	Ldlr
**A5, A6**	Amphiregulin	**D19, D20**	Endoglin	**H1, H2**	Leptin
**A7, A8**	Angiopoietin-1	**D21, D22**	Endostatin	**H3, H4**	Lif
**A9, A10**	Angiopoietin-2	**D23, D24**	Fetuin A (Ahsg)	**H5, H6**	Lipocalin-2
**A11, A12**	Angiopoietin-like 3	**E1, E2**	Fgf-a	**H7, H8**	Lix
**A13, A14**	Baff	**E3, E4**	Fgf21	**H9, H10**	M-Csf
**A15, A16**	Cd93	**E5, E6**	Flt-3 ligand	**H11, H12**	Mmp2
**A17, A18**	Ccl2	**E7, E8**	Gas 6	**H13, H14**	Mmp3
**A19, A20**	Ccl3	**E9, E10**	G-CSF	**H15, H16**	Mmp9
**A21, A22**	Ccl5	**E11, E12**	Gdf15	**H17, H18**	Myeloperoxidase (Mpo)
**A23, A24**	Reference Spots	**E13, E14**	Gm-Csf	**H19, H20**	Osteopontin (Opn)
**B3, B4**	Ccl6	**E15, E16**	Hgf	**H21, H22**	Osteoprotegerin
**B5, B6**	Ccl11	**E17, E18**	Icam-1	**H23, H24**	Pd-Ecgf
**B7, B8**	Ccl12	**E19, E20**	Ifn-γ	**I1, I2**	Pdgf-bb
**B9, B10**	Ccl17	**E21, E22**	Igfbp1	**I3, I4**	Pentraxin 2
**B11, B12**	Ccl19	**E23, E24**	Igfbp2	**I5, I6**	Pentraxin 3
**B13, B14**	Ccl20	**F1, F2**	Igfbp3	**I7, I8**	Periostin
**B15, B16**	Ccl21	**F3, F4**	Igfbp5	**I9, I10**	Pref1
**B17, B18**	Ccl22	**F5, F6**	Igfbp6	**I11, I12**	Proligerin
**B19, B20**	Cd14	**F7, F8**	Il-1α	**I13, I14**	Pcsk9
**B21, B22**	Cd40	**F9, F10**	Il-1β	**I15, I16**	Rage
**C3, C4**	Cd160	**F11, F12**	Il-1ra	**I17, I18**	Rbp4
**C5, C6**	Chemerin	**F13, F14**	Il-2	**I19, I20**	Reg3G
**C7, C8**	Chitinase 3-like 1	**F15, F16**	Il-3	**I21, I22**	Resistin
**C9, C10**	Tissue factor	**F17, F18**	Il-4	**I23, I24**	Reference Spots
**C11, C12**	Complement component C5/C5a	**F19, F20**	Il-5	**J1, J2**	Reference Spots
**C13, C14**	Complement factor D	**F21, F22**	Il-6	**J3, J4**	E-Selectin
**C15, C16**	C-reactive protein	**F23, F24**	Il-7	**J5, J6**	P-Selectin
**C17, C18**	Cx3cl1	**G1, G2**	Il-10	**J7, J8**	SerpinE1 (PAI-1)
**C19, C20**	Cxcl1	**G3, G4**	Il-11	**J9, J10**	SerpinF1
**C21, C22**	Cxcl2	**G5, G6**	Il-12 p40	**J11, J12**	Thrombopoietin
**D1, D2**	Cxcl9	**G7, G8**	Il-13	**J13, J14**	Tim-1
**D3, D4**	Cxcl10	**G9, G10**	Il-15	**J15, J16**	Tnfα
**D5, D6**	Cxcl11	**G11, G12**	Il-17A	**J17, J18**	Vcam-1
**D7, D8**	Cxcl13	**G13, G14**	Il-22	**J19, J20**	Vegf
**D9, D10**	Cxcl16	**G15, G16**	Il-23	**J21, J22**	Wisp-1
**D11, D12**	Cystatin C	**G17, G18**	Il-27 p28	**J23, J24**	Negative Control
**D13, D14**	Dkk-1	**G19, G20**	Il-28A/B		

Table S2. Differentially expressed genes in cells on aged matrix

Upregulated in cells on Aged Matrix					Upregulated in cells on Young Matrix			
	p-value	Adjusted p-value	Fold change			p-value	Adjusted p-value	Fold change
NDRG1	1.89E-73	6.34E-69	6.46		***HSP90AA1***	1.79E-78	5.99E-74	2.18
TXNIP	6.20E-59	2.08E-54	2.36		***HSPA8***	6.30E-59	2.11E-54	1.98
SLC2A1	1.61E-56	5.40E-52	3.04		***NME1***	2.17E-54	7.26E-50	1.88
ADSSL1	1.30E-54	4.35E-50	1.97		***SRM***	4.92E-49	1.65E-44	1.78
CASP14	4.48E-52	1.50E-47	4.21		***CCND1***	4.86E-43	1.63E-38	2.40
EGLN3	5.39E-48	1.81E-43	2.16		***KRT7***	1.10E-37	3.68E-33	2.04
SLC6A8	3.42E-44	1.15E-39	2.56		***AREG***	4.31E-36	1.45E-31	4.78
C4orf3	3.92E-44	1.32E-39	1.81		***KRT18***	2.65E-33	8.90E-29	2.11
P4HA1	1.47E-38	4.94E-34	1.86		***TNFRSF12A***	9.24E-31	3.10E-26	1.96
PNRC1	2.71E-36	9.08E-32	2.02		***RPL22L1***	1.96E-26	6.58E-22	2.04
ZNF395	2.99E-35	1.00E-30	1.85		***KRT81***	1.55E-24	5.21E-20	3.24
AHNAK2	1.52E-34	5.11E-30	1.91		***PHLDA1***	2.16E-23	7.25E-19	1.85
GJA1	2.61E-31	8.75E-27	2.20		***TUBA1B***	2.17E-18	7.29E-14	1.81
CA12	6.42E-30	2.15E-25	2.69		***THBS1***	7.60E-16	2.55E-11	2.13
CD109	2.27E-25	7.63E-21	1.90		***FGFBP1***	9.32E-14	3.13E-09	1.93
MAF	1.45E-23	4.86E-19	2.18		***ALDH1A3***	2.99E-10	1.00E-05	1.92
MALAT1	3.22E-23	1.08E-18	1.92		***TFPI2***	2.45E-08	0.000823	2.25
NEAT1	2.57E-20	8.63E-16	1.82		***KRT6B***	3.09E-08	0.00104	1.94
BTG1	8.57E-20	2.88E-15	1.82					
LOXL2	6.46E-17	2.17E-12	1.86					
DST	6.68E-15	2.24E-10	1.92					
IGFBP3	1.02E-14	3.42E-10	2.18					
MME	2.07E-14	6.95E-10	1.80					
TIMP3	6.41E-14	2.15E-09	2.65					
LOX	8.48E-14	2.84E-09	3.14					
MT1X	1.62E-12	5.44E-08	2.34					
COL17A1	4.24E-11	1.42E-06	1.85					
SERPINE1	3.01E-08	0.00101	2.35					

Table S3. Differentially expressed genes in cluster 7 cells compared to other cell clusters

Upregulated in cluster 7 cells					Downregulated in cluster 7 cells			
	p-value	Adjusted p-value	Fold change			p-value	Adjusted p-value	Fold change
ZEB2	5.26E-111	1.76E-106	9.06		***S100A14***	5.37E-97	1.80E-92	109
ROS1	1.66E-107	5.57E-103	6.58		***FXYD3***	5.55E-87	1.86E-82	83.4
LOX	1.88E-101	6.29E-97	67.2		***ADIRF***	2.77E-81	9.29E-77	153
VCAN	5.13E-97	1.72E-92	50.8		***TACSTD2***	8.75E-80	2.94E-75	77.2
NNMT	1.96E-94	6.58E-90	9.88		***KRT15***	8.81E-78	2.95E-73	151
CHST2	4.04E-94	1.36E-89	12.0		***KRT7***	2.34E-76	7.85E-72	19.2
MME	1.10E-92	3.68E-88	21.3		***RAB25***	5.77E-75	1.93E-70	7.33
SELENOP	2.56E-90	8.60E-86	12.1		***CALML3***	7.20E-74	2.41E-69	29.8
SPARC	3.79E-88	1.27E-83	35.2		***SPINT2***	9.37E-73	3.14E-68	6.26
FN1	6.79E-84	2.28E-79	53.8		***TSPAN1***	8.25E-71	2.77E-66	8.65
C1R	1.70E-83	5.72E-79	4.75		***LAD1***	7.42E-70	2.49E-65	4.19
COL5A2	3.58E-81	1.20E-76	5.95		***AP1M2***	1.15E-68	3.84E-64	3.22
LAMB1	1.22E-78	4.11E-74	11.4		***JUP***	2.26E-65	7.57E-61	6.50
SORBS2	3.82E-77	1.28E-72	12.3		***TINAGL1***	7.78E-65	2.61E-60	6.19
HIPK2	1.24E-76	4.15E-72	9.85		***KRT18***	4.01E-64	1.35E-59	10.8
PNRC1	1.33E-74	4.45E-70	9.63		***PROM2***	8.88E-64	2.98E-59	4.03
HTRA1	3.59E-72	1.20E-67	20.7		***CAST***	4.00E-63	1.34E-58	6.42
LOXL2	8.76E-71	2.94E-66	8.23		***CLTB***	4.42E-63	1.48E-58	6.69
ADPRHL1	3.56E-69	1.19E-64	1.80		***TMEM238***	2.57E-62	8.63E-58	4.06
MFGE8	4.07E-69	1.36E-64	7.81		***LAMB3***	7.76E-60	2.60E-55	13.3
TIMP1	4.42E-69	1.48E-64	14.3		***MAL2***	8.15E-60	2.73E-55	9.16
SNAI2	5.93E-69	1.99E-64	7.96		***IL18***	2.08E-57	6.97E-53	4.44
BTG1	4.25E-67	1.43E-62	8.16		***PHLDA2***	3.11E-57	1.04E-52	10.2
RAB31	6.21E-63	2.08E-58	6.59		***HEBP2***	6.31E-57	2.12E-52	5.33
VKORC1	6.95E-63	2.33E-58	5.19		***CSTB***	1.58E-56	5.28E-52	38.1
APLP2	3.08E-62	1.03E-57	4.12		***C6orf132***	2.13E-56	7.13E-52	8.48
FBN1	6.58E-61	2.21E-56	4.08		***C19orf33***	1.33E-55	4.48E-51	3.41
ZBTB16	3.35E-60	1.13E-55	5.15		***GALNT3***	4.08E-55	1.37E-50	2.76
NRCAM	8.27E-60	2.77E-55	4.00		***CDH1***	5.01E-54	1.68E-49	4.22
FOXO1	9.71E-60	3.26E-55	7.62		***C1orf116***	5.35E-54	1.79E-49	3.30
COL5A1	2.26E-59	7.58E-55	2.31		***ANXA3***	7.86E-54	2.64E-49	6.44
CTSB	3.14E-59	1.05E-54	4.65		***TMEM54***	9.13E-54	3.06E-49	3.23
FKBP5	5.20E-59	1.74E-54	6.67		***SERPINB2***	2.18E-53	7.31E-49	23.9
MALAT1	3.73E-58	1.25E-53	8.12		***ALDH1A3***	2.59E-53	8.69E-49	23.8
NEAT1	9.63E-58	3.23E-53	10.0		***GPX2***	3.79E-53	1.27E-48	10.7
GJA1	2.27E-57	7.61E-53	7.04		***CD24***	1.02E-52	3.41E-48	15.6
LAMC1	8.90E-57	2.99E-52	4.48		***LRRC8A***	1.03E-50	3.44E-46	4.74
MAF	1.88E-53	6.30E-49	10.5		***A2ML1***	1.40E-50	4.70E-46	5.28
SEMA6D	3.73E-53	1.25E-48	2.90		***INPP4B***	1.77E-50	5.93E-46	3.50
RAB13	1.15E-52	3.86E-48	3.90		***PCBD1***	2.36E-49	7.93E-45	2.74
ITGB1	2.46E-52	8.25E-48	3.63		***PERP***	3.67E-49	1.23E-44	5.56
PMP22	3.28E-52	1.10E-47	1.87		***KRT17***	2.49E-48	8.36E-44	16.4
ASPH	1.48E-51	4.98E-47	4.32		***GIPC1***	5.34E-48	1.79E-43	3.07
PSAP	5.13E-50	1.72E-45	3.65		***LMO7***	6.36E-48	2.13E-43	5.45
PDE4DIP	6.47E-50	2.17E-45	4.13		***CITED4***	1.43E-47	4.81E-43	4.61
NREP	1.98E-49	6.64E-45	3.94		***PPL***	1.72E-47	5.77E-43	4.46
MMP7	2.23E-49	7.47E-45	25.7		***FERMT1***	2.46E-47	8.25E-43	5.35
MMP2	3.35E-49	1.12E-44	4.55		***LSR***	3.69E-47	1.24E-42	3.24
CALD1	1.31E-48	4.40E-44	4.57		***LLGL2***	4.51E-47	1.51E-42	2.70

NPR3	2.31E-48	7.73E-44	3.48		***GPRC5A***	1.21E-46	4.05E-42	13.0
ALCAM	5.38E-48	1.80E-43	8.28		***MALL***	1.67E-46	5.59E-42	4.27
VIM	1.21E-47	4.05E-43	7.26		***KLK5***	3.09E-46	1.04E-41	6.13
PIK3R1	6.17E-47	2.07E-42	6.11		***TALDO1***	4.33E-46	1.45E-41	3.22
TREM1	2.41E-46	8.09E-42	4.96		***EFHD2***	1.15E-45	3.86E-41	3.48
XIST	2.47E-46	8.28E-42	3.91		***DMKN***	3.33E-45	1.12E-40	3.17
TSC22D3	3.38E-45	1.13E-40	5.53		***SDCBP2***	5.14E-45	1.72E-40	2.58
TGFBI	3.55E-45	1.19E-40	8.13		***CSTA***	5.18E-45	1.74E-40	8.60
FBXO32	1.15E-44	3.86E-40	18.0		***CD9***	1.39E-44	4.65E-40	5.20
PLOD1	1.48E-44	4.95E-40	3.87		***ANXA1***	2.54E-44	8.52E-40	7.30
LAMP2	3.31E-44	1.11E-39	3.10		***PRRG4***	2.96E-44	9.92E-40	2.73
HCFC1R1	4.24E-44	1.42E-39	4.75		***HSP90AA1***	7.59E-44	2.55E-39	3.80
COPS8	6.04E-44	2.03E-39	4.13		***ST14***	1.13E-43	3.79E-39	3.54
IGF1R	1.17E-43	3.92E-39	3.18		***INAVA***	1.32E-43	4.43E-39	2.07
GPC6	1.86E-43	6.23E-39	2.46		***GSTP1***	2.19E-43	7.34E-39	4.68
PLOD2	2.52E-43	8.46E-39	7.48		***CBLC***	2.80E-43	9.39E-39	1.80
TENT5A	4.07E-43	1.37E-38	6.55		***CCND1***	2.92E-43	9.78E-39	9.35
RUNX2	4.99E-43	1.67E-38	3.39		***RAB27B***	3.13E-43	1.05E-38	2.46
MMP14	6.72E-43	2.25E-38	4.25		***TTC9***	2.33E-42	7.82E-38	4.04
OS9	1.40E-42	4.71E-38	3.02		***CXCL16***	2.58E-42	8.65E-38	2.52
P4HA2	1.80E-42	6.04E-38	3.93		***ELF3***	6.86E-42	2.30E-37	12.5

Table S4. Human cytokines tested in dot blot assay of KTB21 cells seeded on matrices

Spot Location	Protein	Spot Location	Protein	Spot Location	Protein
**A1, A2**	Reference Spots	**D11, D12**	IGFBP2	**G13, G14**	MIF
**A3, A4**	Adiponectin	**D13, D14**	IGFBP3	**G15, G16**	MIG
**A5, A6**	Apolipoprotein A-I	**D15, D16**	IL1α	**G17, G18**	MIP1α/MIP1β
**A7, A8**	Angiogenin	**D17, D18**	IL1β	**G19, G20**	MIP3α
**A9, A10**	Angiopoietin-1	**D19, D20**	IL1ra	**G21, G22**	MIP3β
**A11, A12**	Angiopoietin-2	**D21, D22**	IL2	**G23, G24**	MMP9
**A13, A14**	BAFF	**D23, D24**	IL3	**H1, H2**	Myeloperoxidase
**A15, A16**	BDNF	**E1, E2**	IL4	**H3, H4**	Osteopontin (OPN)
**A17, A18**	Complement component C5/C5a	**E3, E4**	IL5	**H5, H6**	PDGF-AA
**A19, A20**	CD14	**E5, E6**	IL6	**H7, H8**	PDGF-AB/BB
**A21, A22**	CD30	**E7, E8**	IL8	**H9, H10**	Pentraxin 3
**A23, A24**	Reference Spots	**E9, E10**	IL10	**H11, H12**	PF4
**B3, B4**	CD40 ligand	**E11, E12**	IL11	**H13, H14**	RAGE
**B5, B6**	Chitinase 3-like 1	**E13, E14**	IL12 p70	**H15, H16**	RANTES
**B7, B8**	Complement factor D	**E15, E16**	IL13	**H17, H18**	RBP4
**B9, B10**	C-reactive protein	**E17, E18**	IL15	**H19, H20**	Relaxin 2
**B11, B12**	Crypto-1	**E19, E20**	IL16	**H21, H22**	Resistin
**B13, B14**	Cystatin C	**E21, E22**	IL17A	**H23, H24**	SDF1α
**B15, B16**	DKK-1	**E23, E24**	IL18Bpa	**I1, I2**	SERPINE1
**B17, B18**	DPPIV	**F1, F2**	IL19	**I3, I4**	SHBG
**B19, B20**	EGF	**F3, F4**	IL22	**I5, I6**	ST2
**B21, B22**	EMMPRIN	**F5, F6**	IL23	**I7, I8**	TARC
**C3, C4**	ENA-78	**F7, F8**	IL24	**I9, I10**	TFF3
**C5, C6**	Endolgin	**F9, F10**	IL27	**I11, I12**	TfR
**C7, C8**	FAS ligand	**F11, F12**	IL31	**I13, I14**	TGFα
**C9, C10**	FGFb	**F13, F14**	IL32	**I15, I16**	Thrombospondin 1
**C11, C12**	FGF7	**F15, F16**	IL33	**I17, I18**	TNFα
**C13, C14**	FGF19	**F17, F18**	IL34	**I19, I20**	uPAR
**C15, C16**	FLT3 ligand	**F19, F20**	IP10	**I21, I22**	VEGF
**C17, C18**	G-CSF	**F21, F22**	I-TAC	**I23, I24**	Reference Spots
**C19, C20**	GDF15	**F23, F24**	Kallikrein 3	**J1, J2**	Reference Spots
**C21, C22**	GM-CSF	**G1, G2**	Leptin	**J3, J4**	Vitamin D BP
**D1, D2**	GROα	**G3, G4**	LIF	**J5, J6**	CD31
**D3, D4**	Growth Hormone	**G5, G6**	Lipocalin 2	**J7, J8**	TIM3
**D5, D6**	HGF	**G7, G8**	MCP1	**J9, J10**	VCAM1
**D7, D8**	ICAM-1	**G9, G10**	MCP3	**J23, J24**	Negative Control
**D9, D10**	IFN-γ	**G11, G12**	M-CSF		

Table S5. Human cancer-related proteins tested in dot blot assay of KTB21 cells seeded on matrices

Spot Location	Protein	Spot Location	Protein	Spot Location	Protein
**A1, A2**	Reference Spots	**C15, C16**	HER2	**F5, F6**	MMP2
**A3, A4**	α-Fetoprotein	**C17, C18**	HER3	**F7, F8**	MMP3
**A5, A6**	Amphiregulin	**C19, C20**	HER4	**F9, F10**	MMP9
**A7, A8**	Angiopoietin-1	**C21, C22**	FGFb	**F11, F12**	MST1
**A9, A10**	Angiopoietin-like 4	**C23, C24**	-	**F13, F14**	MUC1
**A11, A12**	ENPP2	**D1, D2**	MFH1	**F15, F16**	Nectin 4
**A13, A14**	AXL	**D3, D4**	FKHR	**F17, F18**	Osteopontin
**A15, A16**	BCL2L1	**D5, D6**	Galectin 3 (GAL3)	**F19, F20**	TP27/KIP1
**A17, A18**	CA125	**D7, D8**	GM-CSF	**F21, F22**	TP53
**A19, A20**	E-Cadherin	**D9, D10**	HCG	**F23, F24**	PDGF-AA
**A21, A22**	VE-Cadherin	**D11, D12**	HGF R	**G1, G2**	CD31
**A23, A24**	Reference Spots	**D13, D14**	HIF1α	**G3, G4**	Progesteron R
**B1, B2**	-	**D15, D16**	HNF3β	**G5, G6**	Progranulin
**B3, B4**	CAPG	**D17, D18**	HO-1	**G7, G8**	Prolactin
**B5, B6**	Carbonic Anhydrase IX	**D19, D20**	ICAM-1	**G9, G10**	Prostasin
**B7, B8**	Cathepsin B	**D21, D22**	IL2ra	**G11, G12**	E-Selectin
**B9, B10**	Cathepsin D	**D23, D24**	IL6	**G13, G14**	SERPINB5 (Maspin)
**B11, B12**	Cathepsin S	**E1, E2**	IL8	**G15, G16**	SERPINE1 (PAI-1)
**B13, B14**	CEACAM-5	**E3, E4**	IL18 BPa	**G17, G18**	SNAIL
**B15, B16**	Decorin	**E5, E6**	Kallikrein 3	**G19, G20**	SPARC
**B17, B18**	DKK1	**E7, E8**	Kallikrein 5	**G21, G22**	Survivin
**B19, B20**	DLL1	**E9, E10**	Kallikrein 6	**G23, G24**	Tenascin C
**B21, B22**	HER1	**E11, E12**	Leptin	**H1, H2**	Thrombospondin 1
**B23, B24**	-	**E13, E14**	Lumican	**H3, H4**	TIE2
**C1, C2**	-	**E15, E16**	CCL2/MCP1	**H5, H6**	u-Plasminogen activator (uPA
**C3, C4**	Endoglin	**E17, E18**	CCL8/MCP2	**H7, H8**	VCAM-1
**C5, C6**	Endostatin	**E19, E20**	CCL7/MCP3	**H9, H10**	VEGF
**C7, C8**	Enolase 2	**E21, E22**	M-CSF	**H11, H12**	Vimentin
**C9, C10**	eNOS/NOS3	**E23, E24**	Mesothelin	**I1, I2**	Reference Spots
**C11, C12**	EpCAM	**F1, F2**	CCL3/MIP1α	**I23, I24**	Negative Control
**C13, C14**	ERα	**F3, F4**	CCL20/MIP3α		

Movie S1. Migration of KTB21 cells on the young matrix.
Movie S2. Migration of KTB21 cells on the aged matrix.
Movie S3. Migration of KTB21 cells on the young matrix after incubation in scramble siRNA.
Movie S4. Migration of KTB21 cells on the aged matrix after incubation in scramble siRNA.
Movie S5. Migration of KTB21 cells on the young matrix after LOX knock down.
Movie S6. Migration of KTB21 cells on the aged matrix after LOX knock down.
Movie S7. Migration of MDA-MB-231 cells on the young matrix.
Movie S8. Migration of MDA-MB-231 cells on the aged matrix.
Movie S9. Migration of MDA-MB-231 cells on the young matrix after incubation in scramble siRNA.
Movie S10. Migration of MDA-MB-231 cells on the aged matrix after incubation in scramble siRNA.
Movie S11. Migration of MDA-MB-231 cells on the aged matrix after LOX knock down.
